# Multifunctional Fe-based coordination polymer nano-bomb modified with β-lapachone and CaO_2_ for targeted tumor dual chemodynamic therapy with enhanced ferroptosis and H_2_O_2_ self-supply

**DOI:** 10.1186/s12951-023-02287-2

**Published:** 2024-01-03

**Authors:** Pan Zhao, Liyang Gong, Le Chang, Huiping Du, Meijuan Geng, Siyu Meng, Liangliang Dai

**Affiliations:** 1 Xi’an Key Laboratory of Stem Cell and Regenerative Medicine, Institute of Medical Research, Northwestern Polytechnical University, Xi’an, 710072 China; 2 Shaanxi Provincial Key Laboratory of Infection and Immune Diseases, Shaanxi Provincial People’s Hospital, Xi’an, 710068 China

**Keywords:** Dual-cascade amplification, Chemodynamic therapy, Self-initiated nano-bomb, Fe-based coordination polymer, H_2_O_2_ self-supplying

## Abstract

Chemodynamic therapy (CDT) is seriously limited by the inadequacy of exogenous catalytic ions and endogenous H_2_O_2_ in tumors. Herein, a multifunction nano-bomb integrated with calcium peroxide (CaO_2_) and β-lapachone as donors of H_2_O_2_ and GSH-sensitive Fe-based coordination polymer as provider of catalytic ions was constructed for dual cascade-amplified tumor CDT. This hyaluronic acid (HA)-modified nano-bomb could be specially endocytosed by breast cancer cells through a targeting pathway, degraded and released cargoes in response to the GSH-rich cytoplasm. Furthermore, the released CaO_2_ and β-lapachone could significantly self-generated sufficient H_2_O_2_, which could dual-cascade amplify CDT and induce severe oxidative to tumors via cooperating with the delivered iron ions from nano-bombs. Moreover, the unloaded iron and calcium ions could further accelerate tumor damage by overloading Ca^2+^ and ferroptosis, as accompanied by good magnetic resonance imaging (MRI). In vitro and in vivo studies collectively reveal that this nano-bomb not only self-initiates double cascade-amplified CDT via self-generation of H_2_O_2_, but also efficiently activates ferroptosis and initiates Ca^2+^ overloading, consequently significantly tumor growth suppression. This study offers a novel tumor-initiated nano-bomb for dual cascade-amplified CDT and bioimaging with activated ferroptosis and self-supplying H_2_O_2_.

**Supplementary Information:**

The online version contains supplementary material available at 10.1186/s12951-023-02287-2.

## Introduction

CDT could transform the endogenous H_2_O_2_ to hydroxyl radical (·OH) with higher cytotoxicity via Fenton or Fenton-like reaction and cause effective tumor damage, which is known as a promising anticancer approach [1–4]. Since the Fenton reaction is generally mediated by the H_2_O_2_ and typical catalytic ions (e.g., Fe^2/3+^, Mn^2+^, Cu^2+^, etc.), the dose of two substances is thus closely associated with the efficiency of CDT [5–9]. Therefore, the sufficient supply of H_2_O_2_ and catalytic ions in tumor sites is the key to ensure efficient tumor CDT [10, 11].

Although relatively high H_2_O_2_ concentrations have been reported in the tumor microenvironment, it is far from the amount required to achieve the desired CDT efficiency [12, 13]. Accordingly, transplantation of H_2_O_2_-affording agents in CDT platforms is a frequent strategy to enhance its antitumor effect. Typically, glucose oxidase or β-lapachone (β-lap) could increase endogenous H_2_O_2_ dose via mediating or involving the catalytic reactions with substrates (e.g., glucose) or enzymes (e.g., nicotinamide adenine dinucleotide (NAD)(P)H: quinone oxidoreductase-1, NQO1) in the tumor microenvironment [14, 15], which are used as the natural donors of H_2_O_2_ and potential enhancer of CDT. Nevertheless, almost all of these catalytic reactions need O_2_ to participate directly or indirectly, and the actual hypoxia and continuous depletion of O_2_ further impedes CDT effect [16]. Thus, there is an urgent need to develop a novel CDT nanoplatform with sufficient H_2_O_2_ self-supplement and hypoxia mitigation or O_2_ replenishment capabilities [17].

Calcium peroxide (CaO_2_), known as a safe functional nanomaterial, which could be hydrolyzed to simultaneously produce O_2_ and H_2_O_2_ in response to a weakly acidic water environment [18–20]. Thus, it can frequently be used as a natural supplier of both oxygen and H_2_O_2_ to relieve hypoxia and enhance oxidative damage in tumors [21, 22]. Further, the introduction of CaO_2_ as a donor for O_2_ and another amplifier for H_2_O_2_ in nanoplatforms loaded with a provider of H_2_O_2_ (e.g., β-lap) appears to be expected to cascade enhance CDT therapeutic efficacy. However, the susceptibility and instability of CaO_2_, as well as the poor water solubility and low bioavailability of β-lap, limit the efficiency of continuous O_2_ and H_2_O_2_ supply in situ for tumors [18]. Fe-based coordination polymer are not only rich in catalytic ions Fe^2+/3+^, but also have the advantages of high drug loading and safe delivery of active or unstable substances, which are natural donor of exogenous catalytic ions for initiating CDT, and are also reliable delivery vehicle for the above-mentioned functional agents for reinforcing CDT efficiency [23, 24]. It is worth mentioning that, for the sake of safety and efficacy, Fe-based coordination polymer needs to be further functionalized with tumor targeting, degradable and coating characteristics, so that it can overcome off-target effects and protect CaO_2_ from early hydrolysis upon normal physiological conditions, thereby improving biosafety and eliminating potential side effects.

**Fig. 1:**
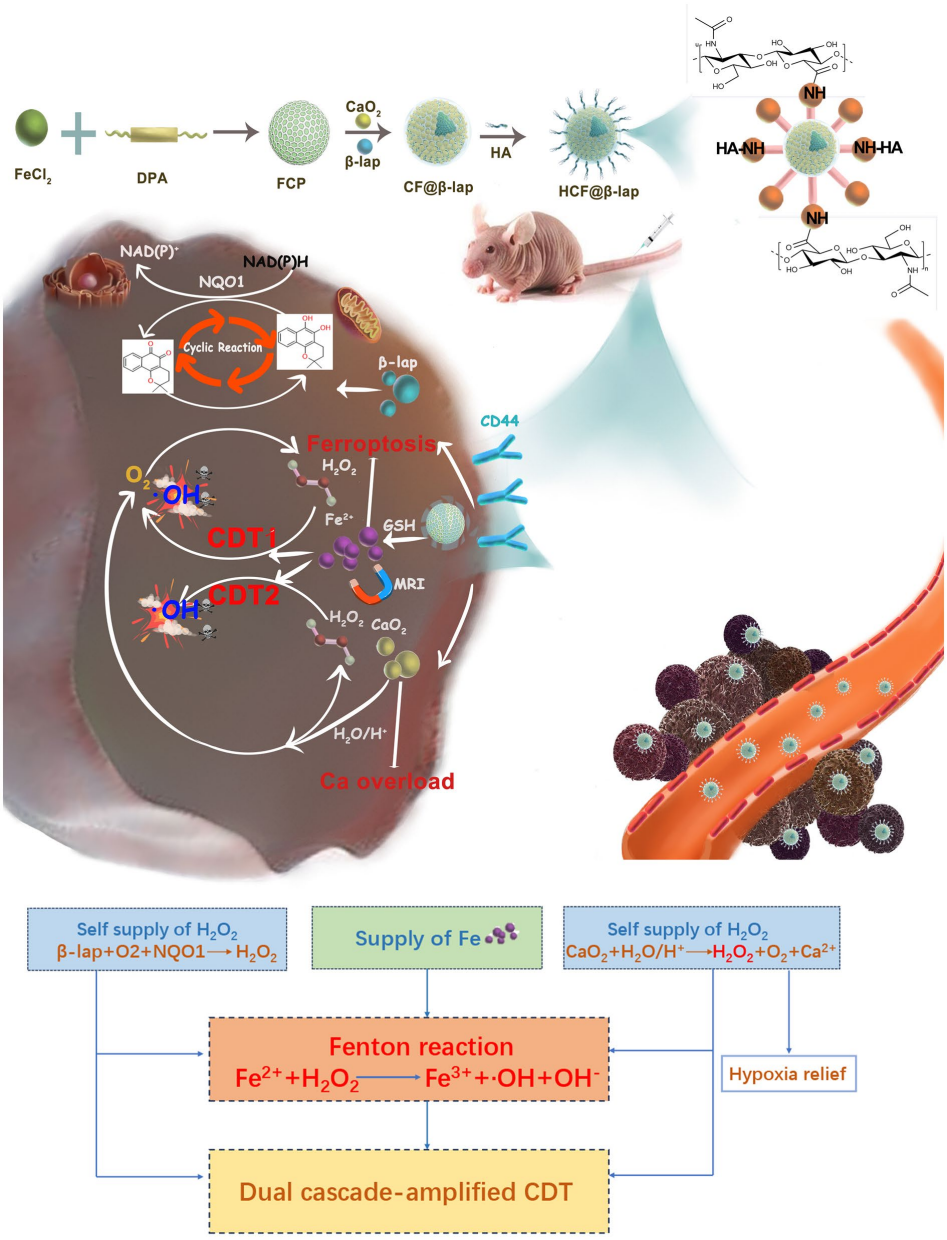
Synthetic path and schematic illustration of HCF@β-lap nano-bomb for dual cascade-amplified CDT and bioimaging with enhanced ferroptosis and H_2_O_2_ self-supply

Herein, a tumor-initiated nano-bomb HCF@β-lap, consisting of the glutathione (GSH)-sensitive Fe-based coordination polymer, CaO_2_ nanoparticles, β-lap and hyaluronic acid (HA, as a tumor-targeting molecule), is constructed for dual-enhanced tumor CDT with endogenous H_2_O_2_ self-supply and O_2_ supplementation (Fig. 1). Taking advantage of CD44 overexpression on the surface of most tumor cells and high levels of GSH in tumor microenvironment [25–28], the nanosystem could be specifically uptake by breast cancer cells and rapidly degrade and release functional cargoes (β-lap, CaO_2_ and Fe^2+^) in response to intracellular GSH. Furthermore, the shed β-lap generates a large amount of H_2_O_2_ through its cyclic reaction involving O_2_ participation. Simultaneously, the collapsed CaO_2_ could rapidly produce H_2_O_2_ and O_2_ via hydrolysis reaction. The endogenous O_2_ self-supply could significantly boost β-lap-based cyclic reaction and accelerate the release of Fe^2+^ and Ca^2+^, the latter of which further reinforce tumor damage via activating ferroptosis and Ca^2+^ overload pathways. More importantly, the H_2_O_2_ dual self-supply generated by the above pathways would remarkably amplify the therapeutic effect of tumor CDT, resulting in the advanced tumor-killing effect in vivo and in vitro. Compared to previous Fe-based coordination polymer, this fabricated nanoplatform has a dual supply of H_2_O_2_ and O_2_ supplement, which significantly improves the efficiency of chemodynamic therapy. Therefore, we develop a tumor-initiated nano-bomb for dual cascade-amplified CDT with activated ferroptosis and self-supplying H_2_O_2_.

## Results and discussion

### Construction and characterization of HCF@β-lap

As for the construction of final nanosystem HCF@β-lap, a facile hydrothermal synthesis method was used to fabricate GSH-sensitive Fe-based coordination polymer by covalently grafting dithiodipropionic acid containing disulfide bonds as linker with Fe^2+^ [29], named as FCP. Secondly, CaO_2_ nanoparticles were synthesized and deposited into the surface of FCP refer to previous protocol [30], the obtained product CaO_2_/FCP was named as CF. Next, β-lap was loaded into above nanoparticles and CaO_2_/FCP@β-lap were harvested (abbreviated as CF@β-lap). Finally, the tumor targeting molecule HA was introduced into the surface of CF@β-lap to form the resultant product HA-CaO_2_/FCP@β-lap (abbreviated as HCF@β-lap) via the chemical conjugation.

**Fig. 2:**
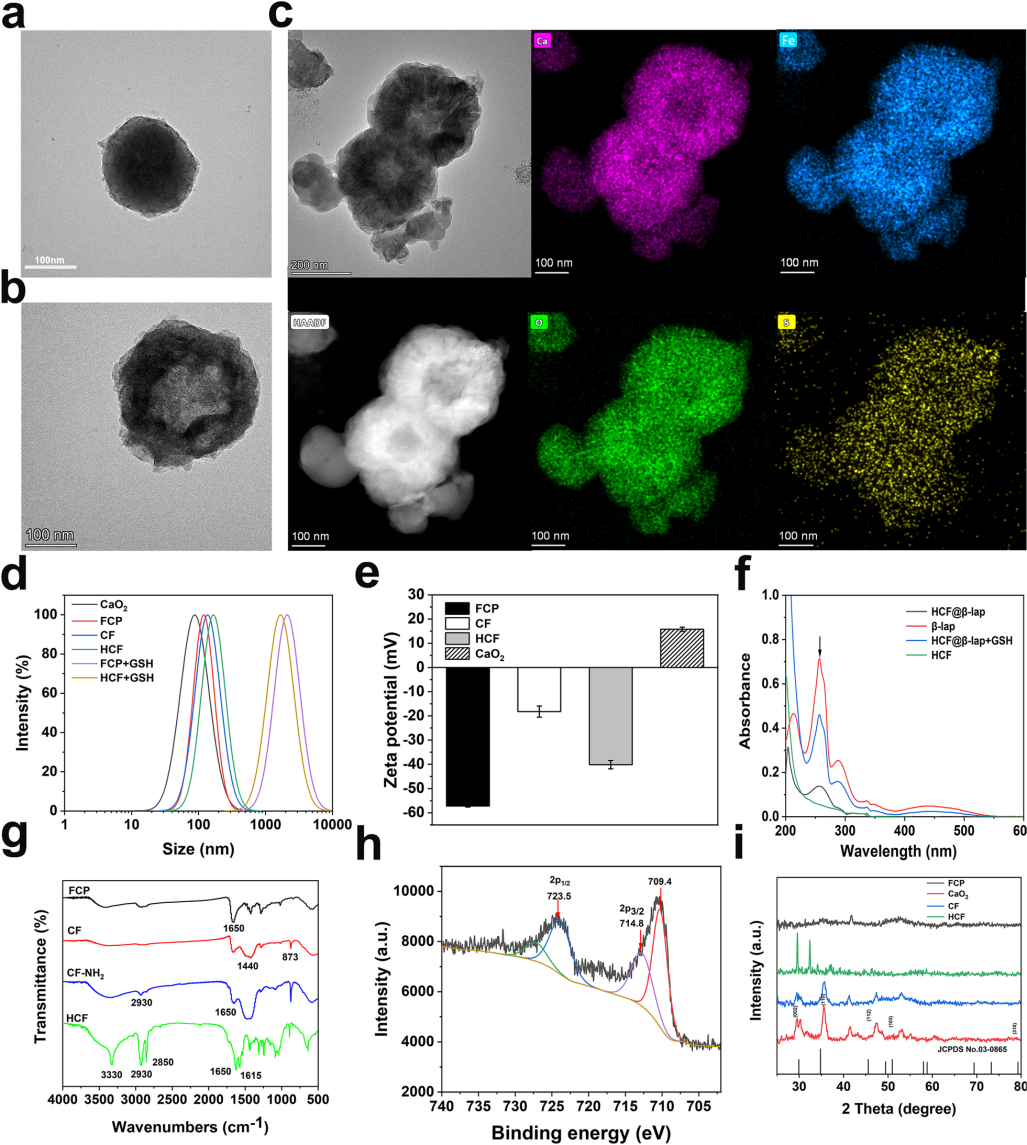
Characterization of HCF@β-lap nanosystem. TEM image of **a** FCP and **b** CF. **c** EDS element mappings of HCF. **d** DLS curves of different nanoparticles with or without GSH treatment. **e** Zeta potential values of FCP, CaO_2_, CF and HCF. **f** Absorbance spectra of HCF, β-lap, HCF@β-lap with or without GSH treatment. **g** FTIR spectra of different treatment groups. **h** XPS spectra and fitted curves of HCF. **i** Powder XRD pattern of various intermediates of HCF.

To verify above synthesis procedures, transmission electron microscopy (TEM) with energy-dispersive X-ray spectroscopy (EDS), dynamic light scattering (DLS), zeta potential, Fourier transform infrared (FTIR) spectra, X-ray photo-electron spectroscopy (XPS), X-ray diffraction (XRD) pattern and ultraviolet-visible (UV-Vis) absorbance spectrum were performed. As shown in Fig. 2a, the prepared FCP, CF and HCF all showed a homogeneous spherical morphology in transmission electron microscopy (TEM) images with the average sizes of 141 nm ± 1.67 nm, 166 nm ± 0.53 nm, and 216 nm ± 2.45 nm (Fig. 2a–c). Notably, the size of nanoparticles was accordingly increase and its structure became blurred with the introduction of CaO_2_ and HA on the surface of HCF (Fig. 2c vs. 2b vs. 2a), which was consistence with the related DLS analysis (Fig. 2d and Additional file 1: Table S1), implying the successful modification of HCF. The EDS result further confirmed the successful synthesis of FCP and functionalization of HCF, shown by the special Fe and S (from FeCl_2_, dithiodipropionic acid), Ca (from CaO_2_) and O elements (from dithiodipropionic acid, CaO_2_, HA) in Fig. 2c. Notably, the optimal mass ratio of CaO_2_ and FCP is 1:1, which was confirmed by the smallest size with the narrow PDI (Additional file 1: Table S2). Furthermore, after conjugation with CaO_2_ and strong negative charged HA, the surface potentials of CF (− 18.4 ± 0.96 mV) and HCF (− 40.13 ± 2.38 mV) were also correspondingly change compared to the natural FCP (− 57.33 ± 2.38 mV) detected by zeta potential measurement (Fig. 2e), confirming again the successful construction of HCF. Additionally, the characteristic absorption peak for β-lap was also demonstrated in the absorption spectrum of HCF@β-lap after β-lap loading, compared to the blank carrier HCF, indicating the successful loading of the ROS donor β-lap with encapsulation content of 30.83% and loading efficiency of 5.81% (Fig. 2f). The characteristic peaks of HA were also found in the FTIR spectra of HCF (Fig. 2g), confirming again the successful construction of HCF. Moreover, the characteristic peaks of Fe 2p1/2 (723.5 eV) and Fe 2p3/2 (709.4 and 714.8 eV) appeared in the XPS of HCF (Fig. 2h), and the peak at 709.4 eV can be attributed to Fe (II), while the peaks at 710.8 and 714.8 eV can be attributed to Fe (III), indicating the successful synthesis of HCF with sufficient content of iron, which could provide sufficient catalytic ions for initiating CDT. Simultaneously, the distinct diffraction peaks of CaO_2_ were all displayed in the XRD spectrums of CF and HCF same to CaO_2_ (Fig. 2i), which was consistence with previous literatures [22, 31], suggesting again the actually conjugation of CaO_2_ and HA. Besides, HCF also exhibited good chemical stability (Additional file 1: Fig. S1), which is reflected in the unchanged crystal structure detected by XRD after 7 days of incubation in physiological environment (pH 7.4) and alkaline condition (0.1 M NaOH). As the degradation of CaO_2_ peroxide induced by acid stimuli, its crystal form is changed accordingly. These results collectively revealed that the successful construction of HCF@β-lap nanosystem.

To evaluate the biostability of HCF@β-lap nanosystem, FCP, CF and HCF@β-lap nanoparticles were respective incubated with 10% serum solution at 37 °C for 6 days, meanwhile, size changes of above two nanoparticles were observed and recorded using DLS. As shown in Additional file 1: Fig. S2, CF showed an overall smaller trend during incubation, which was attributed to the hydrolysis of CaO_2_. While, both the FCP and HCF@β-lap showed negligible size changes upon incubation of 6 days, implying the good biostability. It was benefited from the its strong negative charge feature and shielding effect of HA. This result indicated that the HCF@β-lap nanosystem has good structural stability in serum, thus facilitating drug delivery in vivo. Besides, the consumption capacity of HCF@β-lap nanosystem was investigated by incubating 10 mm GSH with different concentrations of HCF@β-lap (Additional file 1: Fig. S3). It was found that HCF@β-lap can effective consume GSH via the reduction reaction of Fe, which would further contribute to the enhancement of oxidative damage in tumors.

### GSH-responsive disassembly and drug release of HCF@β-lap nanosystem

**Fig. 3:**
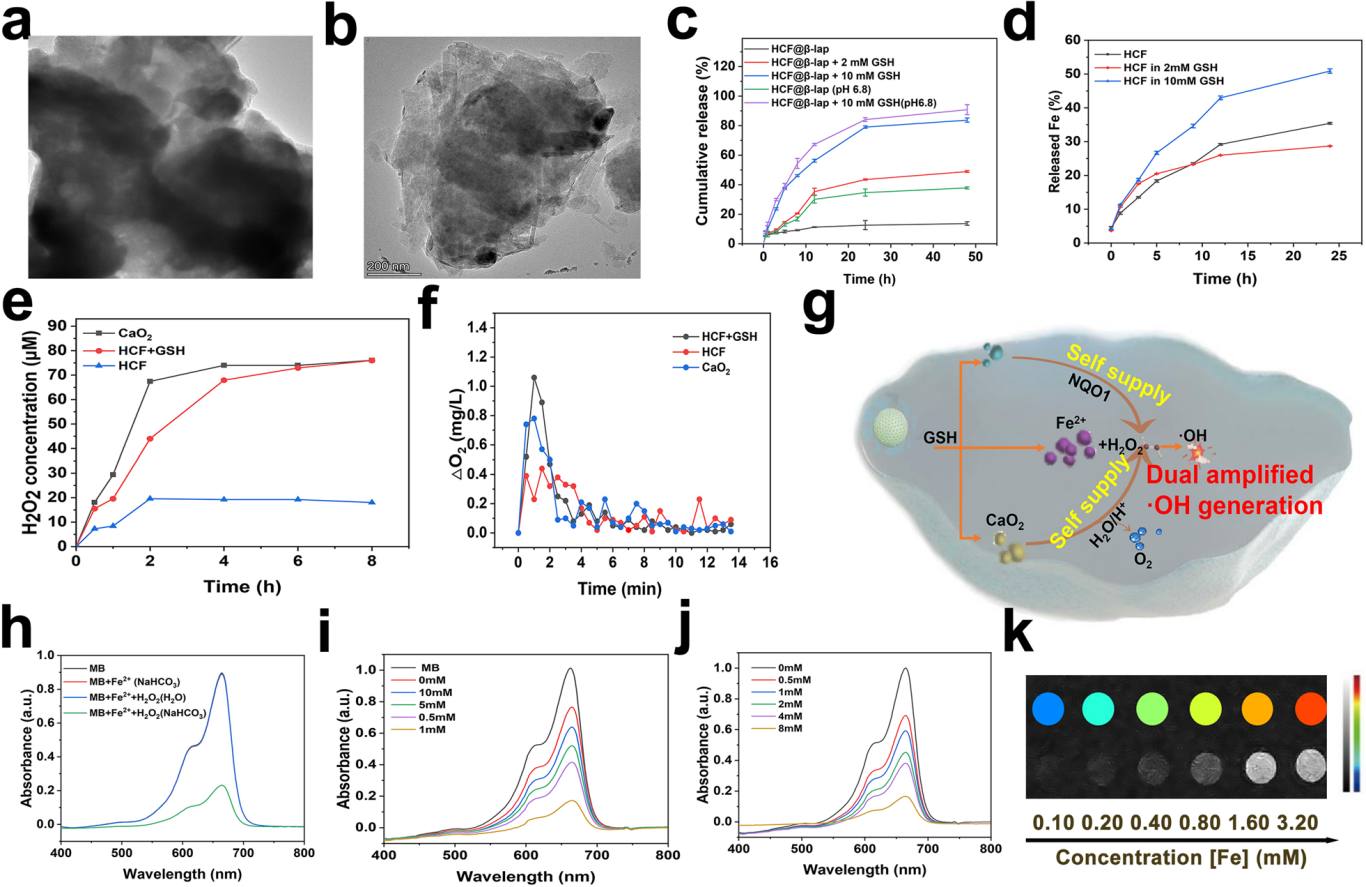
ROS generation and in vitro MRI. TEM images of FCP (**a**) and HCF (**b**) with 10 mM GSH incubation. **c** Cumulative release behavior of β-lap from HCF@β-lap nanosystem after treatment with various concentrations of GSH upon 48 h. **d** The release of Fe element from HCF nanocomposites in different GSH concentrations. **e** H_2_O_2_ cumulative release profile (CaO_2_ = 10 µg mL^−1^). **f** O_2_ concentration measurement in PBS (10 mM, pH = 7.4) (CaO_2_ = 100 µg mL^−1^). **g** Schematic diagram of H_2_O_2_, O_2_ and ·OH generation **h** UV–vis absorption spectra of MB degradation in different solutions [Fe] = 0.5 mM, [H_2_O_2_] = 8 mM, [NaHCO_3_/5% CO_2_] = 25 mM). **i** MB degradation by ·OH generated from different concentration of GSH-treated HCF@β-lap (100 µg mL^−1^) plus H_2_O_2_ (8 mM) and **j** different concentration of H_2_O_2_-treated HCF@β-lap (100 µg mL^−1^) plus GSH (1 mM). [NaHCO_3_/5% CO_2_] = 25 mM. **k** *T*_2_-weighted images

Except for the good biostability, the high-sensitive carriers disassembly and drug release traits were also important for an ideal drug delivery system. To reveal the redox-responsive degradation and drug release behavior of HCF@β-lap nanosystem, 10 mM GSH (stimulation tumor microenvironment) was co-cultured with HCF@β-lap nanosystem for different time intervals, the changes of particle size and morphology and drug release behavior of the nanosystem were monitored by TEM, DLS and UV–vis absorbance spectrum, respectively. As shown in Fig. 3a, b, the morphological structure of both FCP and HCF were obviously changed from an intact spherical shape to a collapsed structure after 4 h of GSH treatment, suggesting the desired disintegration of the nanosystem, which was consistent with DLS data (Fig. 2d and Additional file 1: Table S1). Further, the intensity of the absorption peak of β-lap in the HCF@β-lap nanosystem was increased significantly after incubation with GSH, indicating the indeed release of β-lap (Fig. 2f). The reason could be explained that GSH effectively cleaves the DTA bridging molecule containing disulfide bonds, which in turn causes the disintegration of nano-bomb and the effective drug release.

To further quantitatively monitor the drug release behavior of the nanosystem, on the one hand, we measured the real-time release profiles of β-lap in the HCF@β-lap nanosystem under treatment with different doses of GSH. As shown in Fig. 3c, the drug release from HCF@β-lap is negligible (10%) upon PBS (pH 7.4, stimulation the normal physiological conditions) for 48 h, indicating good encapsulation property. Thus, it does not leak drug into the bloodstream, exhibiting good biostability with reduced side effects. Excitingly, about 43% and 85% of β-lap (nearly complete drug release) were respective release from the HCF@β-lap nanosystem after treatment with 2 mM or 10 mM GSH for 48 h, confirming again the redox-sensitive drug release features of HCF@β-lap nanosystem, which was helpful for achieving the desired tumor killing. The above phenomenon can be explained that GSH could effectively cleave the disulfide bonds linked with irons and dithiodipropionic acid, resulting in rapid disintegration of HCF@β-lap nanosystem and drug release. Notably, the weakly acidic environment can further accelerate the release of drugs under the same condition, as the weak acid stimulus can cause the hydrolysis of CaO_2_ capped on the surface of HCF nanosystem, resulting in an increase in drug release. On the other hand, we also monitored the release of iron ions from HCF@β-lap nanosystem upon different GSH dose. As shown in Fig. 3d, the release of iron ions increases with increasing GSH concentration, confirming again the redox-responsive drug release. Besides, the released iron ions could in turn act as catalysts for the Fenton reaction, catalyzing the conversion of H_2_O_2_ to toxic ·OH, which would further contribute to improving tumor killing efficiency. These result demonstrated that the HCF@β-lap nanosystem exhibited both good biostability under physiological conditions and effectively redox-responsive vehicles disintegration and drug release properties in the tumor microenvironment, displaying the expectant promise for tumor therapy.

### In vitro self-supply H_2_O_2_ and O_2_ and ·OH production

Although HCF@β-lap nanosystem could rapidly release cargoes in response to GSH-rich tumor microenvironment, the actual H_2_O_2_ and O_2_ supplying effect of cargoes (derived from CaO_2_ and β-lap) should be carefully investigated, since it was closely associated the CDT efficacy and following tumor damage. For H_2_O_2_ measurement, on the one hand, the typical Cu (II)-neocuproine spectrophotometric approach was used for calculating the amount of H_2_O_2_ released from CaO_2_ in HCF@β-lap nanosystem. As CaO_2_ nanoparticles could immediately reacted with water to produce H_2_O_2_, the amount of H_2_O_2_ generation was dynamically increase to 67.4 µM over 8 h, while the cumulative H_2_O_2_ in the HCF@β-lap group remained at the same level below 20 µM upon the same time intervals (Fig. 3e), which could be explained that the HA coating layer could actually protected CaO_2_ from prior reaction with water [32], leading to the relatively low H_2_O_2_ generation. Excitingly, the HCF@β-lap regained a rapid H_2_O_2_ production capacity similar to free CaO_2_ in the presence of GSH, resulting from the FCP disassembly and CaO_2_ release triggered by the GSH stimuli. This result suggested that the HCF@β-lap nanosystem exhibited both good biosafety in normal physiological conditions with minimum H_2_O_2_ production and tumor-activated H_2_O_2_ self-production capacity in the GSH-rich tumor microenvironment, which was significantly for boosting CDT efficiency and tumor killing.

On the other hand, the amount of H_2_O_2_ produced by β-lap and CaO_2_ in the HCF@β-lap nanosystem in vitro and the ROS generation by the cascade-amplified CDT were determined by DCFH-DA fluorescent probe. As shown in Additional file 1: Fig. S4a, the blank FCP carrier exhibited negligible green fluorescence, while the strong green fluorescence displayed in β-lap group, confirming the natural H_2_O_2_ supply of β-lap [33]. At same time, CF, CF@β-lap and HCF@β-lap groups also demonstrated the bright green fluorescence, with the sequence CF < CF@β-lap < HCF@β-lap (Additional file 1: Fig. S4b). This observation can be attributed to the following reasons: Firstly, as the natural donors of H_2_O_2_, both CaO_2_ and β-lap could also generate H_2_O_2_ in tumor microenvironment, therefore, the CaO_2_ deposited CF could cause ROS production with the moderate green fluorescence. Secondly, after introduction of β-lap as another H_2_O_2_ donor, CF@β-lap thus displayer higher fluorescence intensity compared to the CF group. Thirdly, benefiting from the targeting effect of HA, HCF@β-lap could be uptake by tumor cells with high efficiency, the abundant CaO_2_ and β-lap was thus accumulated in tumor cells and exhibited the highest fluorescence among all groups. These results further confirmed that HCF@β-lap actually possesses the dual self-supply H_2_O_2_ capacity, displaying the favorable potential for enhanced CDT.

For another thing, the O_2_ production caused by CaO_2_ in HCF@β-lap nanosystem was investigated via a portable real-time dissolved oxygen meter. As demonstrated in Fig. 3f, there was no significant change in O_2_ levels dissolved in HCF@β-lap solution within 13.5 min, indicating the limited O_2_ generation, which was attributed to the coating of HA. In contrast, upon the addition of GSH, the concentration of O_2_ displayed a more rapid increase in the HCF@β-lap solution within the first 1 min, followed by a gradual decline, similar to pure CaO_2_. This phenomenon can be explained by the disintegration of the nanosystem and the exposure of CaO_2_ in response to GSH, followed by rapid hydrolysis of CaO_2_ and the release of O_2_. Notably, the above O_2_ release trend is more pronounced in the presence of weak acidic environment (pH 6.8), which can be attributed to the accelerated hydrolysis of calcium peroxide by the weak acid environment (Additional file 1: Fig. S5). Above findings are strong evidence in support of the capacity of HCF@β-lap to self-generate H_2_O_2_ and O_2_ [19].

As the obvious self-supply of H_2_O_2_ could provide the sufficient fuel for CDT (revealed by Fig. 3g), we subsequently investigate the capacity of ·OH generation using the typical methylene blue (MB) probe. As displayed by Fig. 3h, in the presence of HCO_3_^−^, Fe^2+^ and H_2_O_2_, a large amount of ·OH were generated, which could oxidize MB and transform it into a deep blue complex, resulting in a decrease in absorbance, which was used for characterizing the production of ·OH. Herein, HCF@β-lap nanosystem were incubated with different dose of GSH and MB probe in the presence of NaHCO_3_ to measure the production of ·OH. As shown in Fig. 3i, the absorbance value of MB gradually decreases in this nanosystem as the GSH concentration increase from 0 to 1.0 mM, indicating the ·OH generation. The reason could be explained that iron ions could be quickly released from HCF@β-lap nanoplatform in response to GSH (Fig. 3d), and accompanied by the depletion of GSH and self-generation of H_2_O_2_ (Fig. 3e and Additional file 1: Fig. S3). The iron ions and H_2_O_2_ subsequently participated in the Fenton-like reaction and produced ·OH via CDT, leading to the oxidation of MB and a decrease in absorbance value. Notably, with further increases in GSH concentration (ranged from 1 to 10 mM), the absorbance of MB in above reaction system displayed a surprised increase (Fig. 3j), which was might attributed to the actually scavenging effect of ·OH mediated by the excess GSH [34]. In order to further reveal this explanation, HCF@β-lap were incubated with different concentration of H_2_O_2_ in presence of the fixed GSH concentration (1 mM). The absorbance of MB displayed a H_2_O_2_ dose-dependent decrease tendency (Fig. 3j), proving the effective ·OH production without the absence of excess GSH-induced deletion. These results adequately suggested that HCF@β-lap possessed the tumor-activated self-supply capacity of H_2_O_2_ and O_2_, leading to the effective ·OH production and promising tumor damage.

### Relief hypoxia

As the self-supply of O_2_ from HCF@β-lap not only could enhance the generation of H_2_O_2_ mediated by β-lap, but also play an important role in relieving tumor hypoxia, the levels of hypoxia in 4T1 cells were subsequently monitored after various treatments, using the typically hypoxia detection kit (ROS-ID® Hypoxia/Oxidative stress detection kit, Enzo). As nitric reductase in hypoxic cells could metabolized the probe and exited red fluorescence, thus, there is a positive correlation between the intensity of intracellular red fluorescence and the level of hypoxia. As shown in Additional file 1: Fig. S6, the hypoxia group displayed the obvious red fluorescence, whereas the fluorescence intensity of the HCF@β-lap treatment group decreased significantly, demonstrating the effective hypoxia relief, which could further amplify the self-generation of H_2_O_2_ from HCF@β-lap nanosystem in tumor cells, leading to the enhanced oxidative stress and tumor damage.

### In vitro MRI performance

Considering iron ions was frequently used as contrast agents for MRI applications [35], the redox-responsive HCF@β-lap nanoplatform might possesses MRI performance. To verify this speculation, the longitudinal (*T*_1_) and transverse (*T*_2_) relaxation times of HCF@β-lap with varying iron concentrations (0.10, 0.20, 0.40, 0.80, 1.60, or 3.20 mM) were measured, and the corresponding relaxation rates were calculated. As shown in Additional file 1: Fig. S7, a longitudinal relaxation rate (*r1*) of 0.062 mM^−1^s^−1^ and a transverse relaxation rate (r*2*) of 0.689 mM^−1^s^−1^ was displayed in HCF@β-lap nanoparticles. Since *r1* < *r2*, HCF@β-lap thus can actually serve as a *T*_2_-weighted contrast agent. This was further confirmed by *T*_2_-weighted MRI measurements, where the imaging brightness significantly increased with increasing FCP concentration, resulting in a range of coloration from dark blue to orange (Fig. 3k). Based on these findings, it can be inferred that HCF@β-lap nanosystem not only could rapidly disassembly & release cargo in response to GSH existed in tumor microenvironment, and exhibit the enhanced CDT effects with the dual self-supplying H_2_O_2_ and O_2_ supplement, but also holds good MRI feature.

### Cytotoxicity and the targeted cell uptake of HCF@β-lap

**Fig. 4:**
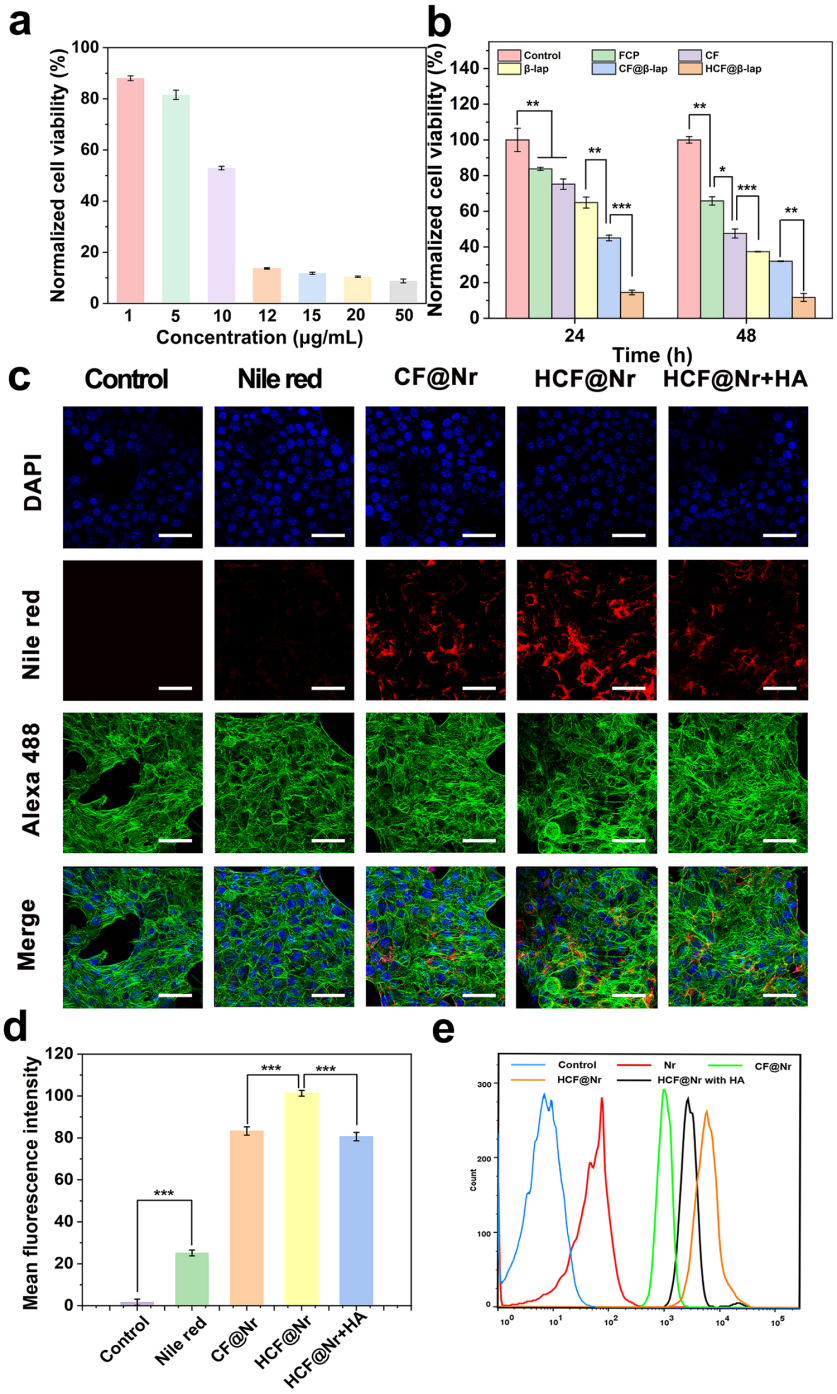
Cytotoxicity and cellar uptake. **a** Cytotoxicity assay of 4T1 cells cultivated with different concentrations of HCF@β-lap. **b** Cytotoxicity assay of 4T1 cells cultivated with different treatment groups for 24 and 48 h, respectively. **c** Cellular uptake and intracellular distribution of different nanosystem in the 4T1 cells after incubation with PBS, Nile Red, CF@Nr, HCF@Nr or HCF@Nr + HA (pretreatment with HA for 2 h) for 24 h, detected by CLSM, the corresponding quantitative analysis of Nr fluorescence (**d**) and FCM (**e**). Nuclei and cytoskeleton were respective stained with DAPI (blue) and Alexa 488-phalloidin (green). Scale bar: 50 μm. Significance analysis was performed using a one-way ANOVA, ***p < 0.001

The biocompatibility and cytotoxicity of HCF@β-lap nanosystem was determined by CCK-8 kit. Upon incubating 4T1 cells with varying concentrations of HCF@β-lap (1, 5, 10, 12, 15, 20, 50 µg/mL) for 24 h, HCF@β-lap nanosystem exhibited dose-dependent cytotoxicity (Fig. 4a), meanwhile, when the concentration of HCF@β-lap was equal to or exceeded 12 µg/mL, the viability of 4T1 cells exhibited a near-complete extinction (about 10.45%). Further, after incubation with different treatment groups (equivalent HCF@β-lap concentration of 12 µg/mL) for 24 h, a noteworthy reduction in cell activity was observed in the HCF@β-lap group, with a percentage decrease of 14.47% (p < 0.001) as compared to the control (99.98%), FCP (83.75%), CF (75.22%), β-lap (64.87%), and CF@β-lap (45.05%) groups (Fig. 4b). After extending the incubation to 48 h, the above tumor death tendency became more obvious. Similar results were further found in live-death experiments (Additional file 1: Fig. S8), confirming again the superior tumour killing effect of the HCF@β-lap nanosystem. The findings indicate a strong likelihood that the HCF@β-lap nanosystem can be effectively internalized by tumor cells and induce tumor killing in vitro with high efficiency.

In order to assess the targeting efficacy of the HCF@β-lap nanosystem towards 4T1 tumor cells, the hydrophobic fluorescent dye Nile Red was incorporated into nanosystem (named as CF@Nr or HCF@Nr) to facilitate the visualization of cellular endocytosis. As shown in Fig. 4c, d, the red fluorescence intensity of HCF@Nr in 4T1 tumor cells was significantly higher than that of CF@Nr (p < 0.001), which was might benefiting from the HA-receptor-mediated endocytosis pathway. To further reveal this mechanism, 4T1 cells were pre-incubated with excess HA molecule for 4 h. The result demonstrated that the intensity of red fluorescence marked with nanoplatform decreased significantly (p < 0.001), indicating weak cellular uptake. The observed outcome can be ascribed to the fact that early incubation of HA competitively occupies the CD44 receptor overexpressed on the surface of tumor cells, resulting in the inability of tumor cells to uptake nanoparticles specifically through the interaction between CD44 and HA-modified HCF nanosystem. The related flow cytometry (FCM) analysis further confirmed the targeted endocytosis pathway of tumor cells toward HCF nanosystem (Fig. 4e).

### Tumor cells apoptosis, Ca^2+^ overload, mitochondrial damage and ferroptosis evaluation

**Fig. 5:**
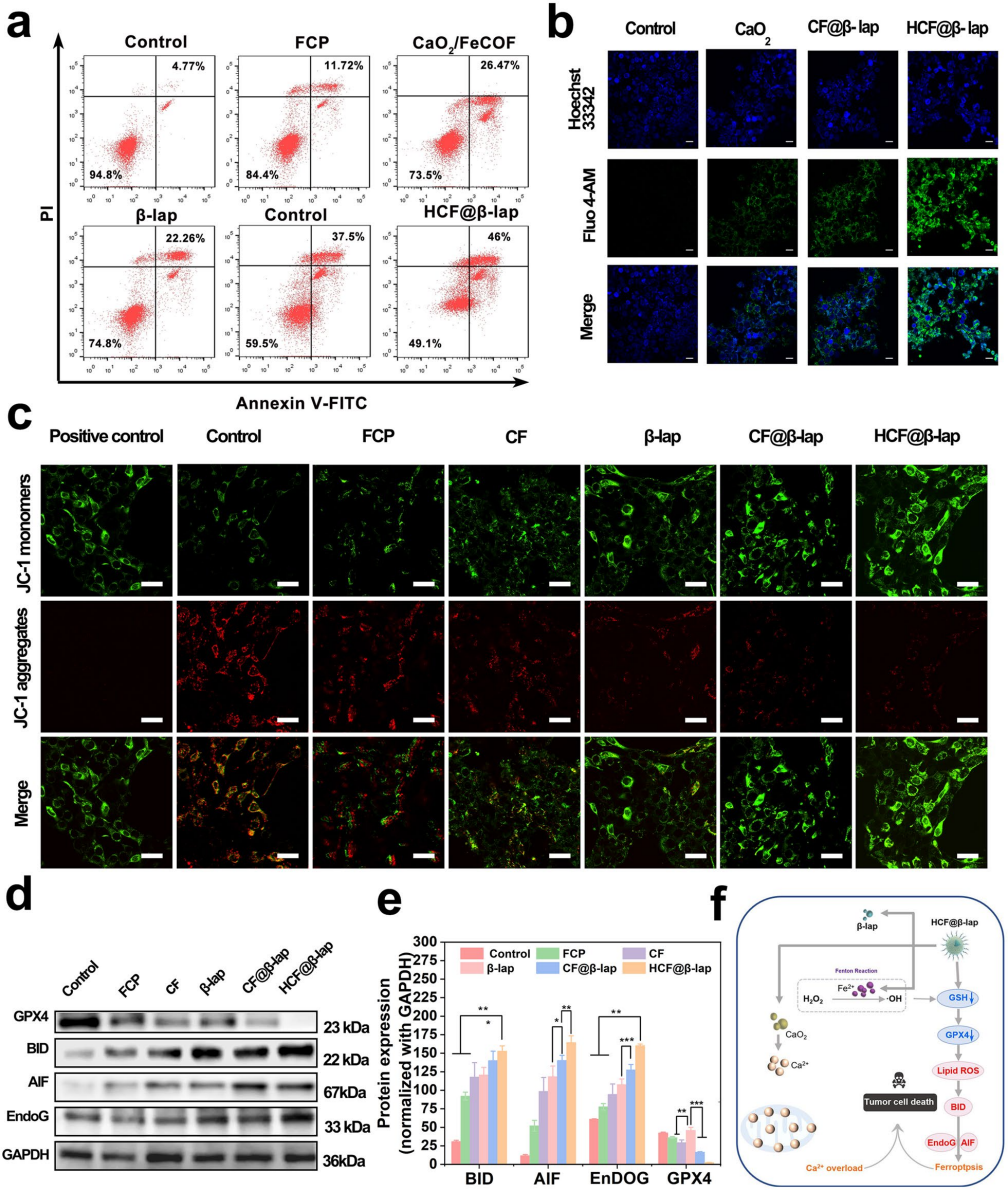
Mitochondrial damage, calcium overload, ferroptosis and apoptosis evaluation. **a** Representative cell apoptosis analysis of 4T1 cells induced by above treatments after 24 h of incubation, detected by FCM. **b** CLSM images of intracellular Ca^2+^ concentration via Fluo-4 AM staining after above treatments. **c** Mitochondrial membrane depolarization of 4T1 cells after treatments with PBS, FCP, CF, β-lap, CF@β-lap and HCF@β-lap for 12 h, detected by CLSM with JC-1 probe staining. **d** The expression level of the ferroptosis related proteins and the corresponding quantitative analysis (**e**) in 4T1 cells after above treatments for 24 h, detected by Western blotting. **f** Schematic illustration of the apoptosis/damage mechanisms of tumor cells induced by HCF@β-lap nano-bomb. Scale bar: 20 μm for **b**, **c**. Significance analysis was performed using a one-way ANOVA, * p < 0.05, **p < 0.01, ***p < 0.001

The viability of cells is intricately linked to either the process of apoptosis or proliferation. The HCF@β-lap nanosystem induces a reduction in cellular viability and facilitates the evaluation of apoptotic or necrotic states. The apoptosis/death degree of 4T1 cells was firstly analyzed using the Annexin V-FITC/PI staining kit. As shown in Fig. 5a and Additional file 1: Fig. S9, benefiting from the respective self-supply of H_2_O_2_ from CaO_2_ and β-lap, both CF and β-lap groups exhibited relatively high apoptosis rate (about 26.5% and 22.3%), which was notably greater than that observed in the blank nanocarrier FCP group (11.72%) and the control group (4.77%). Meanwhile, the integration of dual self-supply of H_2_O_2_ and enhanced CDT strategies resulted in higher cytotoxicity in the CF@L group (37.50%). More importantly, the HCF@β-lap group demonstrated the highest degree of apoptosis and death (approximately 46%), taking advantage of the dual self-supply of H_2_O_2_ and high level of cellular uptake.

Considering HCF@β-lap could also release Ca^2+^ in tumor microenvironment, and the excessive accumulation of Ca^2+^ was positively associated with the programmed cell death, Fluo-4 AM was thus employed to measure the intracellular calcium concentrations and reveal the above apoptosis mechanism. The results depicted in Fig. 5b and Additional file 1: Fig. S10 indicate that the administration of CF@β-lap led to a rise in intracellular Ca^2+^ concentrations in comparison to cells treated solely with CaO_2_. Furthermore, a noteworthy elevation in the concentration of Ca^2+^ was detected in cells treated with HCF@β-lap. This observation is in line with prior findings that the administration of the HCF@β-lap nanosystem to 4T1 cells results in an increased production of ROS, as depicted in Additional file 1: Fig. S4. Hence, an excessive influx of calcium ions leads to heightened levels of oxidative stress and expedites the process of programmed cell death.

To further elucidate the underlying mechanism of HCF@β-lap induced apoptosis, we subsequently investigated the extent of mitochondrial damage in tumor cells. Mitochondrial damage and changes in mitochondrial membrane potential were assessed using the membrane-permeable JC-1 dye. JC-1 dye exhibits red fluorescence when it forms polymers (J-aggregates) in the mitochondrial matrix, indicating normal high mitochondrial membrane potential. Conversely, when the mitochondrial membrane potential is destroyed, JC-1 remains in its monomeric form (J-monomer) and exhibits fluoresces green. In our study, we examined the effect of HCF@β-lap on mitochondrial damage after 12 h pretreatment. As shown in Fig. 5c and Additional file 1: Fig. S11, minimal differences were observed between the control group and the FCP group, indicating that FCP had little impact on mitochondrial damage. In contrast, CaO_2_, β-lap, CF@β-lap and HCF@β-lap all induced varying degrees of mitochondrial damage compared to the control and FCP groups. Among these groups, HCF@β-lap demonstrated the highest level of mitochondrial damage, followed by CF@β-lap and β-lap alone. The results directly indicated that the administration of HCF@β-lap induces impairment of mitochondrial function and perturbation of mitochondrial membrane potential, which serves as an initial biomarker of programmed cell death. The disparities noted in mitochondrial impairment indicate that the HCF@β-lap treatment group is comparatively more efficacious in provoking apoptosis and mitochondrial dysfunction in contrast to the other groups of treatment. The targeting capability of the nanosystem has been observed to improve its bioavailability and induce mitochondrial damage through the enhanced CDT with the dual self-supply of H_2_O_2_, leading to effective elimination of tumor cells and notable anti-tumor effects in vitro.

To comprehensively reveal the mechanism of tumor apoptosis induced by HCF@β-lap nanosystem, the expressions of the related proteins including GPX4, AIF, BID and EndoG were detected using Western blotting (refer to Fig. 5d, e). The findings indicated that the HCF@β-lap group exhibited significantly increased expressions of AIF, BID, and EndoG proteins compared to the control group, indicating the significantly oxidative damage, which was attributed to the dual self-supply of H_2_O_2_ and the cascade-amplified CDT mediated by HCF@β-lap nanosystem. Notably, a noteworthy decrease in GPX4 expression was displayed in HCF@β-lap group, resulting from the depletion of intracellular GSH and activation of ferroptosis. Therefore, taking advantages of the cascade reactions among CaO_2_, β-lap and Fe^2+^ released from HCF@β-lap nanosystem in tumor microenvironment, it could significantly double cascade amplify CDT efficiency, as accompanied by the exciting activation of Ca^2+^ overload and ferroptosis, and ultimately leading to the effective tumor killing (Fig. 5f).

### The anti-tumor effect of HCF@β-lap nanosystem in vivo

**Fig. 6:**
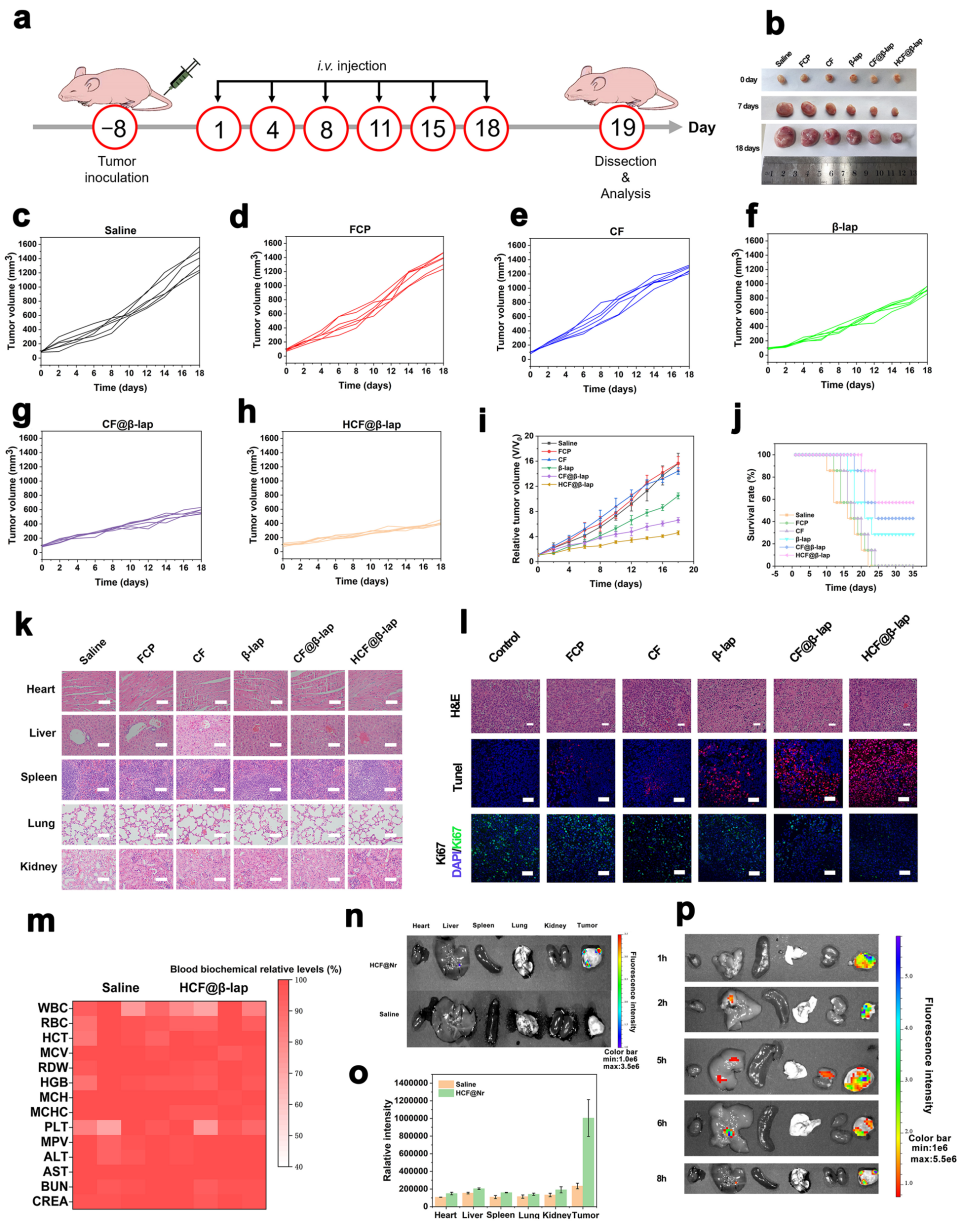
Antitumor effect in vivo, biosafety evaluation and biodistribution. **a** Schematic representation of the protocols of antitumor study in 4T1-bearing BALB/c nude mice. **b** Photographs of xenograft tumor of BALB/c nude mice after treatments with saline, FCP, CF, β-lap, CF@β-lap and HCF@β-lap for 0, 7, and 18 d, respectively. **c**–**h** Tumor volume change curves of the mice in the above treatment groups. **i** Relative tumor volume changes and the average tumor growth curves of the mice after administrations. **j** Survival rate of mice after above administrations. **k** H&E staining images for major tissues. **l** H&E, TUNEL and Ki67 immunofluorescence staining images for tumors. **m** The blood biochemical levels and hematological indices of mice after 24 h of administration. **n** Fluorescence imaging and **o** the corresponding quantitative analysis of the organs of tumor-bearing mice after HCF@Nr administration at 6 h. **p** Fluorescence imaging of the organs of tumor-bearing mice after HCF@Nr administration at 1, 2, 5, 6, 8 h. Scale bar: 50 μm for **k**, **l**. Color bar: 1 × 10^6^–3.5 × 10^6^ for n, 1 × 10^6^–5.5 × 10^6^ for p

To evaluate the anti-tumor effects of the HCF@β-lap nanosystem in vivo, a 4T1 tumor cell BALB/c nude mouse xenograft model was established. The tumor-bearing mice were injected intravenously with different nanosystem formulations twice a week for 18 days (Fig. 6a). All treated groups exhibited varying degrees of tumor suppression compared to the control group (saline). As shown in Fig. 6b, the rate of tumor development in the FCP group was similar to that in the control group, and the other groups showed better tumor suppression. Tumor volume analysis demonstrated that the HCF@β-lap group had the most successful tumor inhibition effect and no significant loss of animal body weight (Fig. 6c–i and Additional file 1: Fig. S12), which could be attributed to dual CDT and superior tumor targeting. In addition, HCF@β-lap treatment also effectively extended the survival time of tumor-bearing mice to a high survival rate of 35 days (Fig. 6j), suggesting a practical anti-tumor effect in vivo. In order to evaluate the biosafety of the HCF@β-lap nanosystem, we monitored the changes in body weight and histological characteristics of the primary tissues of tumor-bearing mice and after different groups. As expected, the significant tissues (i.e., heart, liver, spleen, lung and kidney) of the HCF@β-lap group had normal and intact histological structures, similar to those of the control group (Fig. 6k), again showing good biocompatibility.

As shown in Fig. 6l and Additional file 1: Fig. S13, H&E staining, TUNEL, and Ki67 were further used to detect apoptosis and proliferation inhibition in vivo. HCF@β-lap induced the most severe tumor damage, as indicated by the apparent tissue separation in the H&E images and a large number of magenta dots in the TUNEL observations. Furthermore, IFC analysis of Ki67 in tumor tissues confirmed that HCF@β-lap effectively down-regulated Ki67 expression, displaying its superior anti-tumor effects again. Finally, the routine blood tests and biochemical analyses were performed on the tumor-bearing mice after 24 h and 7 day of administration for further evaluating the biosafety of nanosystem. As shown in Fig. 6m and Additional file 1: Fig. S14, there were also no significant changes in the routine blood and physiological and biochemical parameters in the HCF@β-lap group compared to the control group (saline). These results confirmed the good in vivo biosafety of the HCF@β-lap nanosystem. Subsequently, the in vivo pharmacokinetics and biological dispersion of the nanosystem evaluation were explored further to verify the targeting of the nanosystem. On the one hand, HCF@β-lap exhibited a longer cycle time compared to CF@β-lap, resulting from the shielding effect of HA (Additional file 1: Fig. S15). On the other hand, after injecting saline and the nanosystem into the tail vein for 6 h, a high number of HCF nanosystem labelled with Nile red were distributed in the tumor lesions and a small number were disseminated in other normal organs (Fig. 6n, o), and the amount of HCF nanosystem enriched in tumor tissues was much higher than in other normal tissues, regardless of the time interval (1–8 h) after dosing treatment (Fig. 6p), confirming again the good tumor targeting effect in vivo.

### In vivo MRI

**Fig. 7:**
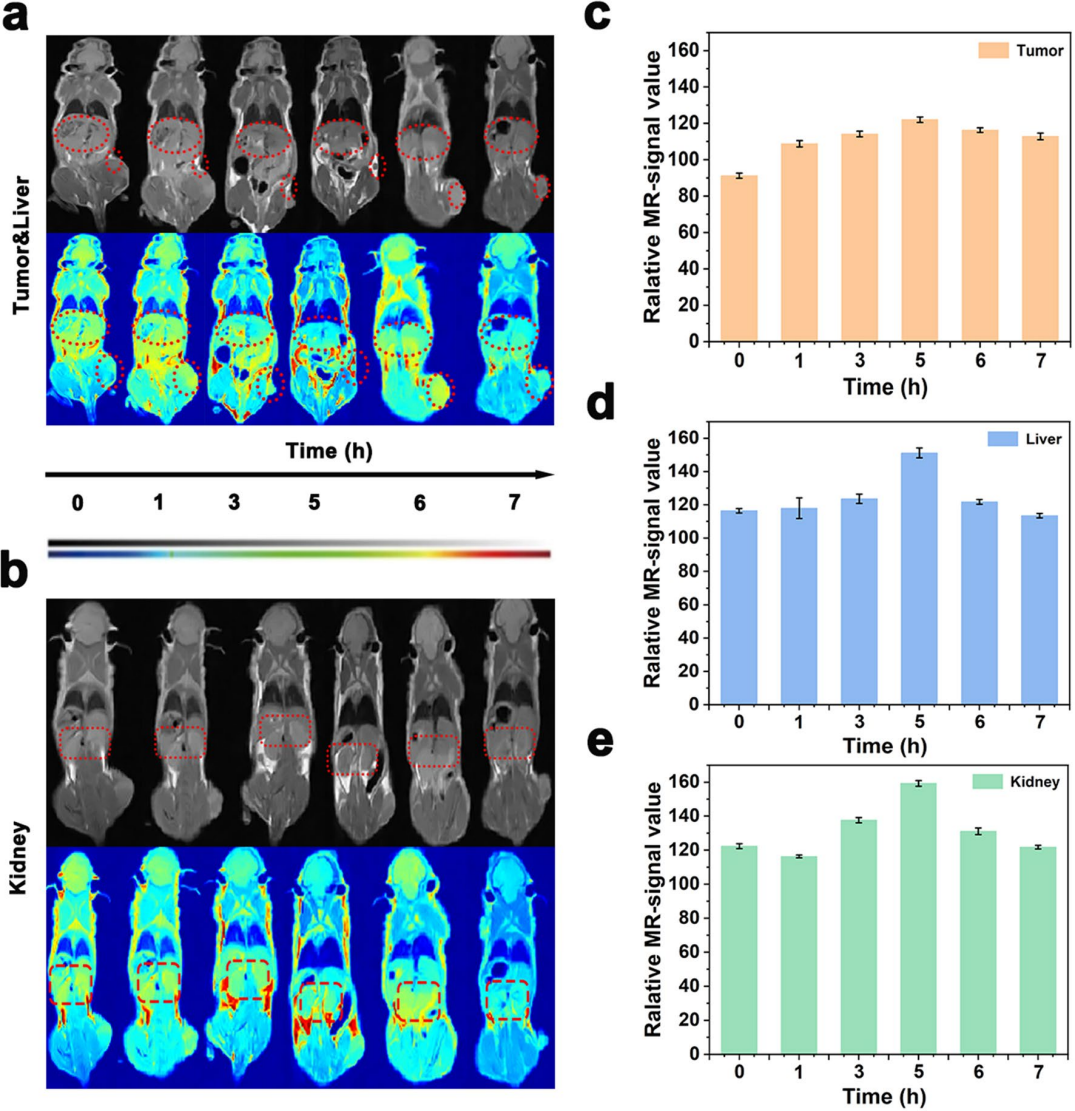
MRI images of the HCF@β-lap in vivo. **a** MRI images of the tumor (indicated with small ellipses) and liver (indicated with large ellipses). **b** MRI images of the kidney (indicated with rectangles). **c**–**e** Corresponding MRI signal intensities for the images shown in **a**, **b**

Inspired by the favorable results of *T*_2_-weighted MRI in vitro, we further investigated the imaging performance of HCF@β-lap in vivo. Firstly, background images of breast cancer tumor-bearing mice were obtained, and then 3.2 mM concentration of Fe (20 mg/kg body weight) was injected into the tail vein, and images were collected at 1, 3, 5, 6, 7 h after injection. As shown in Fig. 7a, the signal at the tumor site brightened and then darkened over time, with the brightest signal observed after 5 h due to the maximum accumulation of HCF@β-lap at the tumor site after 5 h, further evidenced by the biological distribution of HCF@β-lap in major organs (Fig. 7a, b). Subsequently, the nanosystem were gradually metabolized, and the signal at the tumor site became weaker. The statistical results of MRI signal intensity at tumor sites were consistent with imaging results, indicating that HCF@β-lap had good *T*_2_-weighted imaging performance for tumors (Fig. 7c). The relative MRI signal strength of the liver and kidney showed a consistent trend over time (Fig. 7d, e), indicating some retention of nanosystem in the liver and kidney. These results suggested that HCF@β-lap nanosystem could be used as effective *T*_2_-weighted MRI contrast agents for tumor-specific diagnosis.

## Conclusions

In short, a GSH-sensitive FCP-based nanosystem functionalized with donors of O_2_ and H_2_O_2_ (CaO_2_ and β-lap) was designed for dual cascade-amplified tumor CDT with H_2_O_2_ self-supply and hypoxia relief features. Briefly, this nanosystem capped with targeting molecule HA could be specially internalized by cancer cells, disassembled and release cargoes in response to the abundant GSH within the cytoplasm. Furthermore, the released CaO_2_ and β-lap can sequentially double self-produce H_2_O_2_ through a cyclic reaction, and along with the production of O_2_, which not only can input enough “fuel” for CDT, but also relieve tumor hypoxia, resulting in severe tumor oxidative damage with the help of sufficient exogenous Fe^2+^ self-supplied by nanosystem. Moreover, the released Ca^2+^/Fe^2+^ can additionally exacerbate tumor damage by activating calcium overload and ferroptosis pathways, and display good MRI imaging capability. Taking advantages of the cascade reactions among CaO_2_, β-lap and Fe^2+^ released from HCF@β-lap nanosystem in tumor microenvironment, the HCF@β-lap could significantly double cascading amplify CDT efficiency and relief hypoxic restriction, as accompanied by the exciting activation of Ca^2+^ overload and ferroptosis, and ultimately causing effective anti-tumor effects with good MRI imaging in vivo and in vitro. It provided a novel tumor-initiated nano-bomb platform to dual enhance tumor CDT efficiency.

## Materials and methods

### Materials

N-hydroxy succinimide (NHS) and 3,3′-dithiodipropionic acid (DPA) were purchased from Sigma-Aldrich (Beijing, China). *N*,*N*-Dicyclohexylcarbodiimide (DCC) were purchased from Alfa Aesar (Shanghai, China). Hyaluronic acid (HA) was purchased from Macklin (Shanghai, China). *N*,*N*-Dimethylformamide (DMF) and dimethyl sulfoxide (DMSO) were purchased from Aladdin (Shanghai, China), β-lapachone (abbreviated as β-lap) was provided from yuanye Bio-Technology (Shanghai, China). Primary antibodies including rabbit anti-BID, rabbit anti-AIF, rabbit anti-EndoG, mouse anti-GAPDH and mouse anti-GPX4 were brought from Proteintech (Wuhan, China). Cell counting kit-8 (CCK-8) was purchased from Sevenbio (Beijing, China). Hoechst 33342 staining solution and mitochondrial membrane potential assay kit with JC-1 were purchased from Beyotime Biotechnology (Shanghai, China), reactive oxygen species assay kit and Fluo-4 AM probe were brought from Solarbio Science & Technology Co., Ltd. (Beijing, China).

### Synthesis of GSH-responsive FCP nanoparticles

Briefly, 107 µL of FeCl_2_ solution (50 mg/mL, dissolved in DMF), 347 µL of DPA containing GSH-sensitive disulfide bonds (15 mg/mL, dissolved in DMF), 300 mg of polyvinylpyrrolidone (PVP-K30) and 263 µL of triethylamine were dissolved in 12.3 mL DMF/Ethanol (V/V, 4/6) and mixed well. Next, the mixture was transferred to a reaction kettle and reacted at 150 °C for 24 h. Finally, the reaction solution was centrifuged (12,000 rpm, 10 min) and washed with ethanol, the GSH-responsive FCP was collected via vacuum drying.

### Synthesis of CaO_2_ nanoparticles

Typically, 300 mg of CaCl_2_, 1.5 mL of ammonia (1 M), 12 mL of PEG 400 and 3 mL of ultrapure water were mixed in a round bottom flask. After stirring well, 1.75 mL of H_2_O_2_ (30%) was added dropwise to the above mixture over 50 min and stirred at room temperature for 2 h. Subsequently, the pH of the mixture was adjusted to 11.5, the precipitate was washed with 0.1 M NaOH and distilled water until the pH reached 8.4. The precipitate was then dried in vacuum at 80 °C, resuspended in anhydrous ethanol and filtered through a 0.45 μm microporous membrane. Finally, the filtrate was collected and dried under vacuum to obtain CaO_2_ nanoparticles.

### Synthesis of CF@β-lap

Briefly, 2 mg of β-lap and 10 mg of FCP were dissolved in 10 mL of methanol. This mixture was stirred for 36 h in the absence of light. The β-lap loaded FCP were obtained through vacuum drying. Subsequently, above nanoparticles and CaO_2_ were mixed with different mass ratio in anhydrous ethanol, and stirred at room temperature for another 12 h. Afterward, the solution was centrifuged (12,000 rpm, 10 min) and the precipitate was collected. After vacuum drying, FCP nanoparticles grafted with CaO_2_ and loaded with β-lap was harvested and named as CF@β-lap.

### Synthesis of HCF@β-lap

Firstly, 10 mg of CF@β-lap was resuspended in 10 mL of DMSO dissolved with DCC (6 mM) and NHS (6 mM) and stirred for 1.5 h. Subsequently, ethylenediamine (7 mM) was added to the above solution and stirred for another 24 h. Above mixture was centrifuged and collected the precipitate (named as CF-NH_2_), which was then resuspended in methanol. At same time, 0.25 mmol HA, 1 mmol DCC and 1 mmol NHS were dissolved in 9 mL methanol and stirred for 1.5 h. Afterward, above precipitate resuspended in methanol was added and stirred for 24 h. Finally, this mixture was centrifuged, the HA modified product HA-CaO_2_/FeCP@β-lap was obtained under vacuum and named as HCF@β-lap.

### Materials characterization

The functionalization procedures of HCF@β-lap and the structure of the various intermediates were characterized by transmission electron microscopy (TEM), energy dispersive spectrometer (EDS), dynamic light scattering (DLS), X-ray photoelectron spectroscopy (XPS, Aglient-5110, USA) and Fourier infrared spectroscopy (FTIR, model 6300, BioRad Co. Ltd., USA). The morphology and physical/chemical properties of FCP-based nano-bomb were characterized by TEM (Talos F200X TEM, FEI), DLS and zeta potential analyzer (Brookhaven 90 Plus PALS), X-ray diffraction (XRD, D8 DISCOVER A25) and UV/vis spectrophotometer (Hitachi U-3900).

### Biostability assay of HCF@β-lap nanosystem

Typically, FCP, CF and HCF@β-lap were incubated with serum for different time points (0, 6, 24, 48, 72, 96, 120 and 144 h), samples were then taken and its size change were monitored by DLS.

### Drug release behavior of HCF@β-lap nanosystem

Briefly, 2 mg of HCF@β-lap nanoparticles were dissolved into 5 mL of PBS buffer (pH 7.4) containing different concentrations of GSH (0, 2 and 10 mM). The mixture was then transferred to a centrifuge tube and placed at 37 °C environment with continuous shaking. At specific time intervals (0.5, 1, 3, 5, 8, 12, 24 and 48 h), 200 µL of the supernatant was removed for measurement and replenished with an equivalent amount of the fresh solution. The amount of drug release is calculated by detecting the absorption peak of the β-lap at 260 nm and its standard curve.

Typically, drug loading content (DLC) and drug loading efficiency (DLE) were calculated using the following formulas:

$$\mathrm{DLC}\; (\%) = \mathrm{Amount}\; \mathrm{of}\; \mathrm{loaded}\; \mathrm{drug}/\mathrm{Weight}\; \mathrm{of}\; \mathrm{drug}\; \mathrm{loaded}\; \mathrm{nanoparticles} \times {100\%},$$

$$\mathrm{DLE}\; (\%) = \mathrm{Amount}\; \mathrm{of}\; \mathrm{loaded}\; \mathrm{drug}/\mathrm{Weight}\; \mathrm{of}\; \mathrm{drug}\; \mathrm{in}\; \mathrm{feed} \times {100\%}.$$

### Fe iron release experiment

HCF (1 mg) was respective dissolved in PBS (pH 7.4) mixtures with different concentration GSH (0, 2 and 10 mM), which were then dispersed in dialysis bags (MWCO 1000) and further immersed in 30 mL of PBS (pH 7.4) bath at 37 °C. At determined time intervals (0, 1, 3, 5, 9, 12, 24 h), a portion (1 ml) of the reaction solution was sampled and supplied with same volume reaction solution, above samples were then analyzed for iron concentration by ICP-AES (America-Aglient-5110).

### Extracellular measurement of O_2_ concentration

Briefly, 3 mL of methanol solution (1 mg/mL) dissolved CaO_2_, HCF, or HCF + GSH (10 mM) were respective added to 27 mL of PBS (pH 7.4) and stirred vigorously at room temperature. At determined time intervals, the real time O_2_ concentration of the solution were monitored using a portable dissolved oxygen meter (JPBJ-608, Rex, INESA Scientific Instruments).

### Extracellular measurement of H_2_O_2_ production

Briefly, 1 µL of CaO_2_ (1 mg/mL) or HCF (equivalent CaO_2_ concentration) or HCF + GSH (10 mM), 49 µL of PBS (pH 7.4), 25 µL of CuSO_4_ (0.01 M) and 25 µL of neocuproine (0.01 M) were added sequentially to the 96-well plates. The 96-well plate was then shaken at room temperature. At the indicated times, the absorbance was measured at 450 nm using a microplate reader. Meanwhile, the standard curve for H_2_O_2_ was obtained.

### Extracellular ·OH production

FCP (100 µL, 2 mg/mL), NaCl (200 µL, 50 mM) and different concentrations of GSH were added to 25 mM NaHCO_3_/5% CO_2_ buffer solution (0, 0.5, 1.0, 5 or 10 mM), keeping the total volume of the solution at 800 µL. The mixed solution was then shaken at 37 °C for 15 min. After centrifugation, MB (100 µL, 100 µg/mL) and H_2_O_2_ (100 µL, 80 mM) were added to the supernatant and incubated for 30 min at 37 °C. The absorbance of MB at 664 nm was finally measured.

### Cell culture and cytotoxicity assay

The 4T1 tumor cell line was purchased from the Cell Bank of the Chinese Academy of Sciences (Shanghai). 4T1 cells were incubated in RPMI-1640 medium containing 10% fetal bovine serum and 1% penicillin-streptomycin with 5% CO_2_ at 37 °C.

For cytotoxicity evaluation, 4T1 cells cultured in 24-well plates were treated with PBS, FCP, CF, β-lap (5 µM), CF@β-lap, HCF@β-lap (206.54 µg/mL, equivalent of 5 µM β-lap) for 24 and 48 h, respectively. Then, the culture solution was discarded and replaced with CCK-8 working solution (Beyotime). The cell activity was calculated according to the protocols recommended by the manufacturers.

### In vitro cellular uptake

4T1 cells seeded on confocal culture dishes or six-well plates were treated with Nr, CF@Nr, HCF@Nr, HCF@Nr with or without HA pretreatment (5 mg/mL, 2 h) for 24 h.

For CLSM analysis, cells cultured in confocal dishes were washed with cold PBS and fixed in 4% paraformaldehyde at 4 °C for 0.5 h. Subsequently, Triton and CellLightTalin-GFP were added to permeate the cell membranes and labeled the cytoskeleton, respectively. Finally, the level of uptake of nanosystem by 4T1 cells was observed using CLSM (FV3000, Olympus, Japan).

For FCM analysis, cells seeded on 6-well plates were digested with EDTA-free trypsin and collected by centrifugation. The cells were then resuspended with an appropriate amount of PBS solution, filtered and analyzed directly by FCM (BD, Biosciences).

### Cell apoptosis assay

Briefly, 4T1 cells seeded on 6-well plates were incubated with above treatment groups for 24 h. Afterward, the cells were harvested via centrifugation and stained by the Annexin V-FITC/PI kit (Beyotime, China) according to the instructions. Finally, the cell samples were examined by FCM was used to measure the apoptosis level of tumor cells.

### Detection of intracellular ROS production

4T1 cells inoculated on confocal culture dishes were treated PBS, FCP, CF, β-lap (15 µM), CF@β-lap and HCF@β-lap (206.54 µg/mL, same dose as β-lap) for 12 h, respectively. After washing off the residual nanomaterial with PBS, DCFH-DA probe (Beyotime, China) was added and incubated for 30 min according to the instructions. After removing the medium, the fluorescence images marked with ROS generation were recorded with CLSM.

### Intracellular oxygen detection

1 × 10^5^ cells/mL of 4T1 cells were first added to a petri plate before being separated into three groups for various treatments: (1) The regular oxygen group, the cells were cultivated with regular oxygen levels; (2) hypoxia group, adherent cells were grown in an anoxic chamber containing 1% O_2_, 5% CO_2_, and 94% N_2_ for 4 h; (3) The adherent cells were firstly cultivated in anoxic chamber for 4 h, followed by treatment with HCF@β-lap for another 4 h in RPMI-1640 media. Above cells were then stained with the hypoxia probe (0.5 µL, 1 mM) (ENZO) for 30 min, washed with PBS for three times, and imaged by CLSM.

### Intracellular Ca^2+^ levels measurement

Typically, 4T1 cells seeded on confocal dishes was cultured with PBS, CaO_2_, CF@β-lap and HCF@β-lap for 12 h, respectively. After removal of the medium, cells were stained with Fluo-4 AM (4 µM) probe according to standard methods. Finally, intracellular Ca^2+^ levels were measured using CLSM.

### Mitochondrial damage

Briefly, the 4T1 cells were respective treated with PBS, FCP, CF, β-lap, CF@β-lap and HCF@β-lap for 12 h. Then, cells were washed with PBS and stained with the JC-1 probe from mitochondrial membrane potential assay kit (Beyotime), according to the instructions. Finally, the degree of mitochondrial damage was evaluated with CLSM.

### Western bolt assay

Briefly, 4T1 cells inoculated on 6-well plates were cultured with above treatment groups with for 24 h. Cell samples were then washed with PBS and lysed with lysate agent. The concentration of total protein was determined using BCA protein assay kit (Beyotime). The expression of target proteins was monitored by western blotting and an imaging system (Vilber fusion FX6. EDGE), according to the protocols recommended by the manufacturers. Quantitative analysis of the data was performed using ImageJ software.

### In vitro antitumor assay

BALB/c female nude mice (5–6 weeks) were purchased from the Institute of Drug Control, Beijing. All animal experiments were carried out in strict accordance with the guidelines of the Institutional Animal Care and Use Committee of Chinese. 100 µL of saline containing 1 × 10^6^ 4T1 cells were injected into the right flank of the BALB/c mice. When the tumor volume reached about 100 mm^3^, the mice were randomly divided into six groups and given intravenous injection with saline, FCP, CF, β-lap, CF@β-lap and HCF@β-lap at a dose of 10 mg/kg β-lap equivalent. The above doses were administered twice a week for 18 days. Meanwhile, body weight and tumor volume were recorded every 2 days. The tumor volume (V) was calculated as V = L×W^2^/2 (L and W are the maximum and minimum dimension of the tumor).

### Blood safety study

HCF@β-lap and saline were injected intravenously into the mice, respectively. After administration for 24 h, the blood of mice was obtained to measure the blood biochemical levels and hematological parameters according to the manufacturer’s protocol.

### H&E, TUNEL and immunofluorescence staining assays

The tumor-bearing mice were euthanized at the end of the studies. The major organs and tumors of the mice were collected, fixed, embedded, sectioned and stained with hematoxylin and eosin for H&E histological study. Meanwhile, tumor sections were also deparaffinized and stained with an in-situ apoptosis detection kit (Beyotime) for TUNEL apoptosis analysis. In addition, de-paraffinized tumor sections were also stained with Ki67 primary antibody and FITC-labelled fluorescent secondary antibody for immunofluorescence staining analysis.

### In vivo MRI performance

In vivo *T*_2_-weighted MRI was conducted on a 0.5 T MRI instrument (MiniMR-60, Niumag), and the 4T1 tumor-bearing BALB/c nude mice were used in the experiments. Briefly, coronal scanning was performed before and after the injection of the HCF@β-lap (0, 1, 3, 5, 6, and 7 h). The dosage of FCP was 100 µL (5 mM). The in vivo *T*_2_-weighted MRI parameters included a field of view of 80 × 80 mm, a TR of 500 ms, a TE of 18.125 ms, a slice thickness of 3 mm.

### Biodistribution in vivo

Briefly, saline and HCF@Nr were injected intravenously into the tumor-bearing mice at a dose of 0.5 mg/kg. 6 h after administration, the tumor-bearing mice in the different treatment groups were euthanized and the heart, liver, spleen, lungs, kidneys and tumors were removed, the small animal live imaging system (PerkinElmer IVIS Lumina LT Series III) was used for photographic observation.

### Statistical analysis

The statistical analysis was performed using the OriginPro software (version 2022) by the student’s t-test and one-way analysis of variance (ANOVA). The data were expressed as means ± standard deviation (SD). The confidence levels of 95% and 99% were regarded as the significant difference.

## Supplementary Information

**Additional file 1: Figure S1.** XRD patterns the HCF upon treatment in different solvents for 7 days. **Figure S2.** Particle size changes of FCP, CF and HCF nanosystem. **Figure S3.** The amount of GSH in the supernatant after 24 hours of reaction between HCF@β-lap at different concentrations and 10 mM GSH in mixed solution. **Figure S4.** Intracellular ROS levels and cell membrane staining. **Figure S5.** O_2_ concentration measurement in the presence of  the weak acidic microenvironment. **Figure S6.** CLSM images of hypoxia level in 4T1 cells after PBS and HCF@β nanosystem treatment for 4 h. **Figure S7.** Relaxation rates *r1* and *r2* of solutions of the HCF@β-lap. **Figure S8.** 4T1 cell live/dead staining after various treatments. The red signal denoted dead cells, while the green signal denoted live cells. **Figure S9.** Quantitative apoptosis statistics of 4T1 cells induced by difference treatments after 24 h of incubation. **Figure S10.** The corresponding quantitative analysis of intracellular Ca^2+^ concentration via Fluo-4, AM staining. **Figure S11.** The relative fluorescence density analysis of 4T1 cells on mitochondrial damage after administration for 12 h using JC-1 probe. **Figure S12.** Changes in mice body weight during administration. **Figure S13.** Tumor-related quantitative analysis of TUNEL and Ki67 immunofluorescence images. **Figure S14.** The blood biochemical levels and hematological indices of mice after 7 day of administration. **Figure S15.** The pharmacokinetics of HCF@β-lap and CF@β-lap.

## Data Availability

All data generated and analyzed during this research are included in this published article and additional file.
