# Correction: Multifunctional Fe-based coordination polymer nano-bomb modified with β-lapachone and CaO_2_ for targeted tumor dual chemodynamic therapy with enhanced ferroptosis and H_2_O_2_ self-supply

**DOI:** 10.1186/s12951-025-03917-7

**Published:** 2026-01-28

**Authors:** Pan Zhao, Liyang Gong, Le Chang, Huiping Du, Meijuan Geng, Siyu Meng, Liangliang Dai

**Affiliations:** 1 Xi’an Key Laboratory of Stem Cell and Regenerative Medicine, Institute of Medical Research, Northwestern Polytechnical University, Xi’an, 710072 China; 2 Shaanxi Provincial Key Laboratory of Infection and Immune Diseases, Shaanxi Provincial People’s Hospital, Xi’an, 710068 China

**Correction: Journal of Nanobiotechnology (2024) 22:3**

10.1186/s12951-023-02287-2

In this article Fig. 6 appeared incorrectly and have now been corrected in the original publication. For completeness and transparency, the correct and incorrect versions are displayed below.

Incorrect Fig. 6


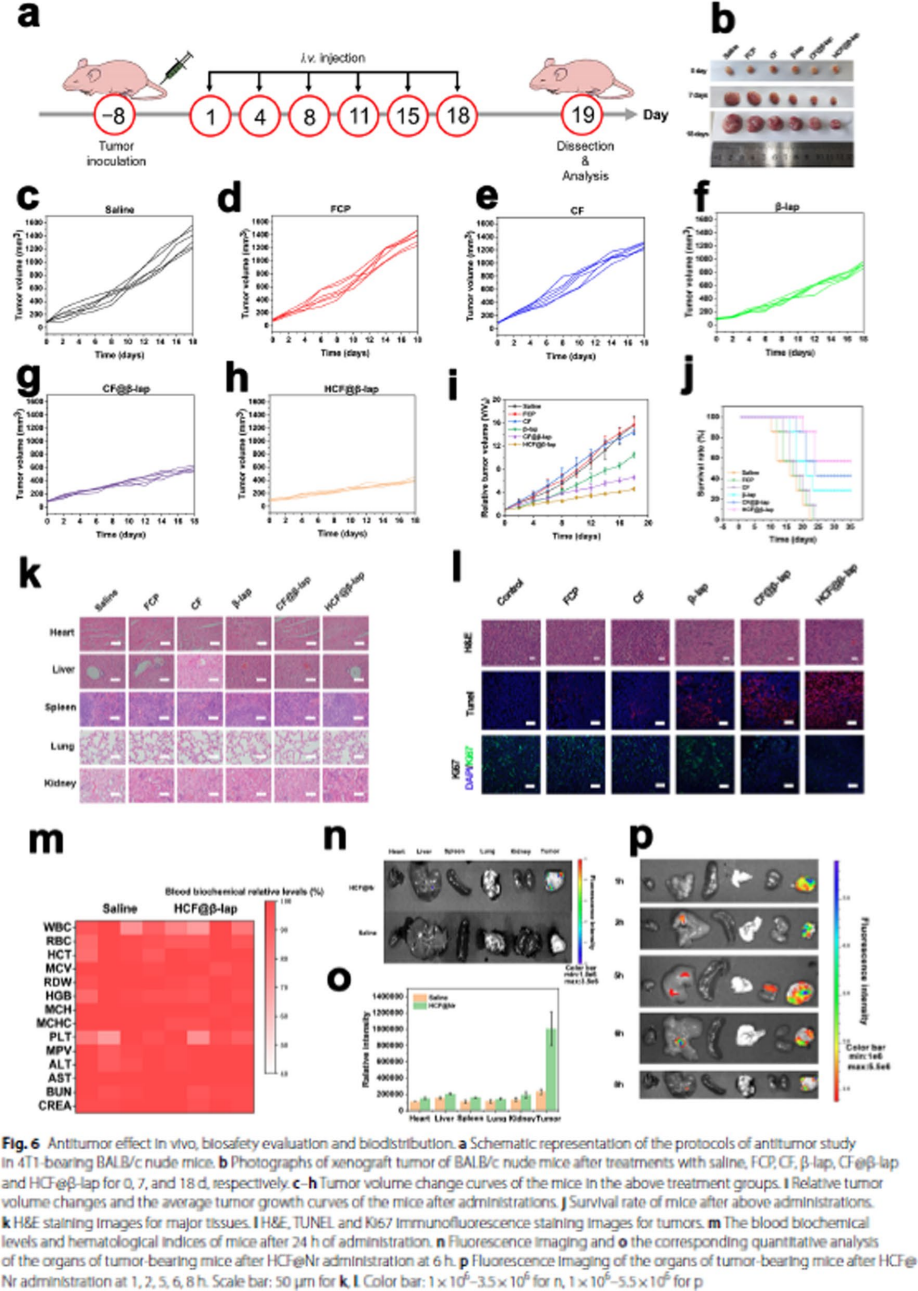


Correct Fig. 6.

**Fig. 6:**
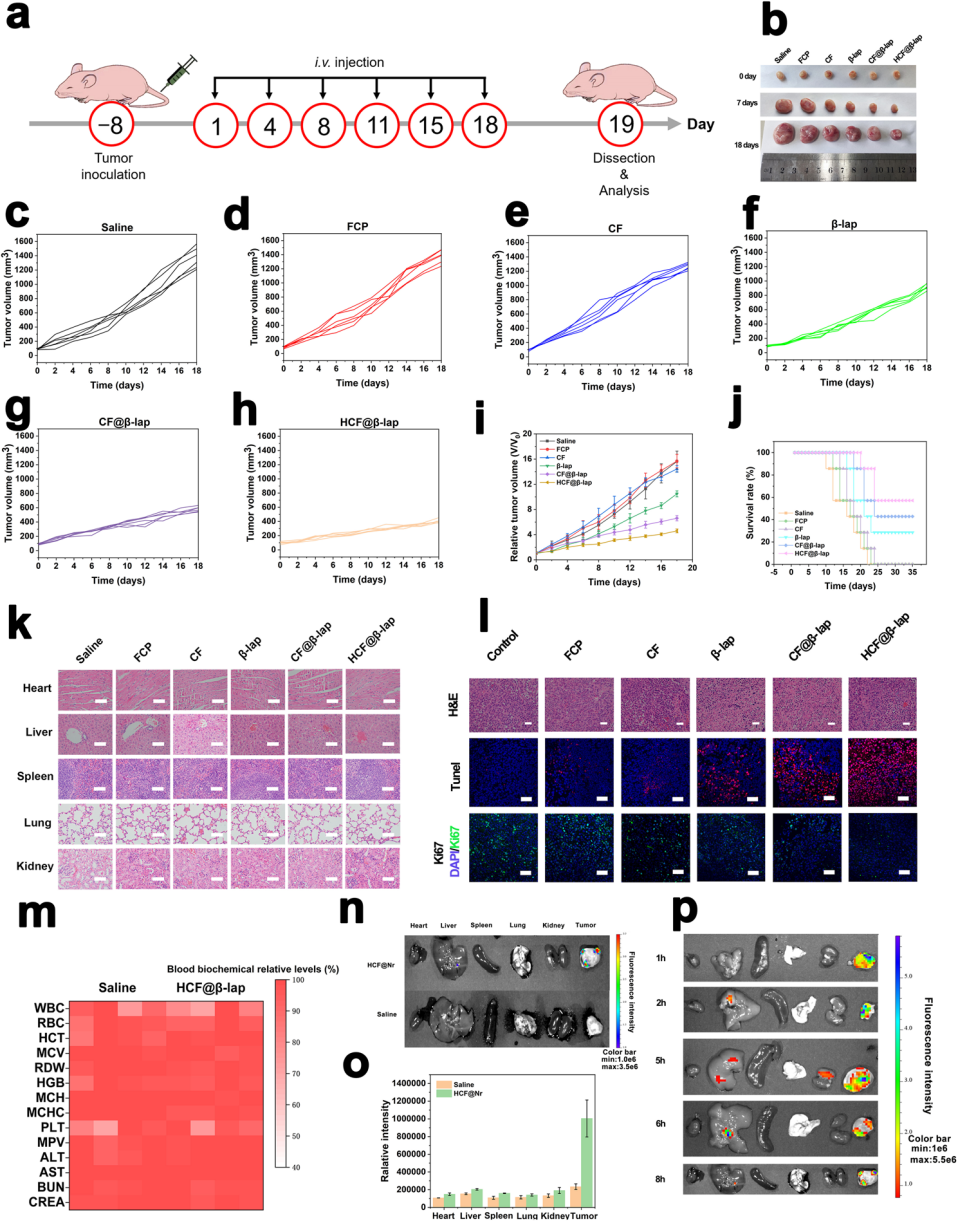
Antitumor effect in vivo, biosafety evaluation and biodistribution. **a** Schematic representation of the protocols of antitumor study in 4T1-bearing BALB/c nude mice. **b** Photographs of xenograft tumor of BALB/c nude mice after treatments with saline, FCP, CF, β-lap, CF@β-lap and HCF@β-lap for 0, 7, and 18 d, respectively. **c**–**h** Tumor volume change curves of the mice in the above treatment groups. **i** Relative tumor volume changes and the average tumor growth curves of the mice after administrations. **j** Survival rate of mice after above administrations. **k** H&E staining images for major tissues. **l** H&E, TUNEL and Ki67 immunofluorescence staining images for tumors. **m** The blood biochemical levels and hematological indices of mice after 24 h of administration. **n** Fluorescence imaging and **o** the corresponding quantitative analysis of the organs of tumor-bearing mice after HCF@Nr administration at 6 h. **p** Fluorescence imaging of the organs of tumor-bearing mice after HCF@ Nr administration at 1, 2, 5, 6, 8 h. Scale bar: 50 μm for **k**, **l**. Color bar: 1 × 10^6^−3.5 × 10^6^ for n, 1 × 10^6^−5.5 × 10^6^ for p

The original article has been corrected.

