# Supplementary material for: Multifunctional Fe-based coordination polymer nano-bomb modified with β-lapachone and CaO2 for targeted tumor dual chemodynamic therapy with enhanced ferroptosis and H2O2 self-supply
Source: J Nanobiotechnology. 2024 Jan 3;22:3. doi: 10.1186/s12951-023-02287-2 (PMC10763286; doi:10.1186/s12951-023-02287-2)
Supplement: Supplementary file 1 — Additional file 1: Figure S1. XRD patterns the HCF upon treatment in different solvents for 7 days. Figure S2. Particle size changes of FCP, CF and HCF nanosystem. Figure S3. The amount of GSH in the supernatant after 24 hours of reaction between HCF@β-lap at different concentrations and 10 mM GSH in mixed solution. Figure S4. Intracellular ROS levels and cell membrane staining. Figure S5. O2 concentration measurement in the presence of the weak acidic microenvironment. Figure S6. CLSM images of hypoxia level in 4T1 cells after PBS and HCF@β nanosystem treatment for 4 h. Figure S7. Relaxation rates r1 and r2 of solutions of the HCF@β-lap. Figure S8. 4T1 cell live/dead staining after various treatments. The red signal denoted dead cells, while the green signal denoted live cells. Figure S9. Quantitative apoptosis statistics of 4T1 cells induced by difference treatments after 24 h of incubation. Figure S10. The corresponding quantitative analysis of intracellular Ca2+ concentration via Fluo-4, AM staining. Figure S11. The relative fluorescence density analysis of 4T1 cells on mitochondrial damage after administration for 12 h using JC-1 probe. Figure S12. Changes in mice body weight during administration. Figure S13. Tumor-related quantitative analysis of TUNEL and Ki67 immunofluorescence images. Figure S14. The blood biochemical levels and hematological indices of mice after 7 day of administration. Figure S15. The pharmacokinetics of HCF@β-lap and CF@β-lap. [file 12951_2023_2287_MOESM1_ESM.docx]

Additional file information for

**Multifunctional Fe-based coordination polymer nano-bomb modified with β-lapachone and CaO_2_ for targeted tumor dual chemodynamic therapy with enhanced ferroptosis and H_2_O_2_ self-supply**

*Pan Zhao ^1^, Liyang Gong ^1^, Le Chang ^2^, Huiping Du ^1^, Meijuan Geng ^1^, Siyu Meng ^1^, Liangliang Dai ^1*^*

^1^ Xi’an Key Laboratory of Stem Cell and Regenerative Medicine, Institute of Medical Research, Northwestern Polytechnical University, Xi’an 710072, China

^2^ Shaanxi Provincial Key Laboratory of Infection and Immune Diseases, Shaanxi Provincial People’s Hospital, Xi’an 710068, China

*Corresponding author: [liangliangdai@nwpu.edu.cn](mailto:liangliangdai@nwpu.edu.cn) (L.L. Dai)

***Lift of Contents***

**Fig. S1**  XRD patterns the HCF upon treatment in different solvents for 7 days………………......S3

[**Fig.** **S2** Particle size changes of FCP, CF and HCF nanosystem. S3](#_Toc113910200)

**Fig. S3**  The amount of GSH ……………………………………………………….…………..S4

**Fig. S4** Intracellular ROS levels and the corresponding quantitative analysis……………………S4

[**Fig. S5** O_2_ concentration measurement in PBS (pH=6.8) ................................................................S5](#_Toc113910201)

[**Fig. S6** CLSM images of hypoxia level in 4T1 cells. S5](#_Toc113910202)

[**Fig. S7**Relaxation rates r1 and r2 of solutions of the HCF@β-lap. S6](#_Toc113910202)

[**Fig. S8** 4T1 cell live/dead staining after various treatments. S6](#_Toc113910203)

[**Fig. S9** Quantitative apoptosis statistics of 4T1 cells induced by difference treatments. S7](#_Toc113910204)

[**Fig. S10** Corresponding quantitative analysis of intracellular Ca^2+^ concentration. S7](#_Toc113910205)

[**Fig. S11** The relative fluorescence density analysis of 4T1 cells on mitochondrial damage. S8](#_Toc113910206)

[**Fig. S12** Changes in mice body weight during administration. S8](#_Toc113910207)

[**Fig. S13** Tumor-related quantitative analysis of TUNEL and Ki67 immunofluorescence images. S9](#_Toc113910208)

**Fig.S14** The blood biochemical levels of mice after 7 day of administration …………………….S10

**Fig. S15** The pharmacokinetics of HCF@β-lap and CF@β-lap...................................................S10

**Table 1** Hydrated particle size and dispersion coefficient of each group……………………...S11

**Table 2** Size, zeta potential and dispersion coefficient at different feed ratios…………………S11


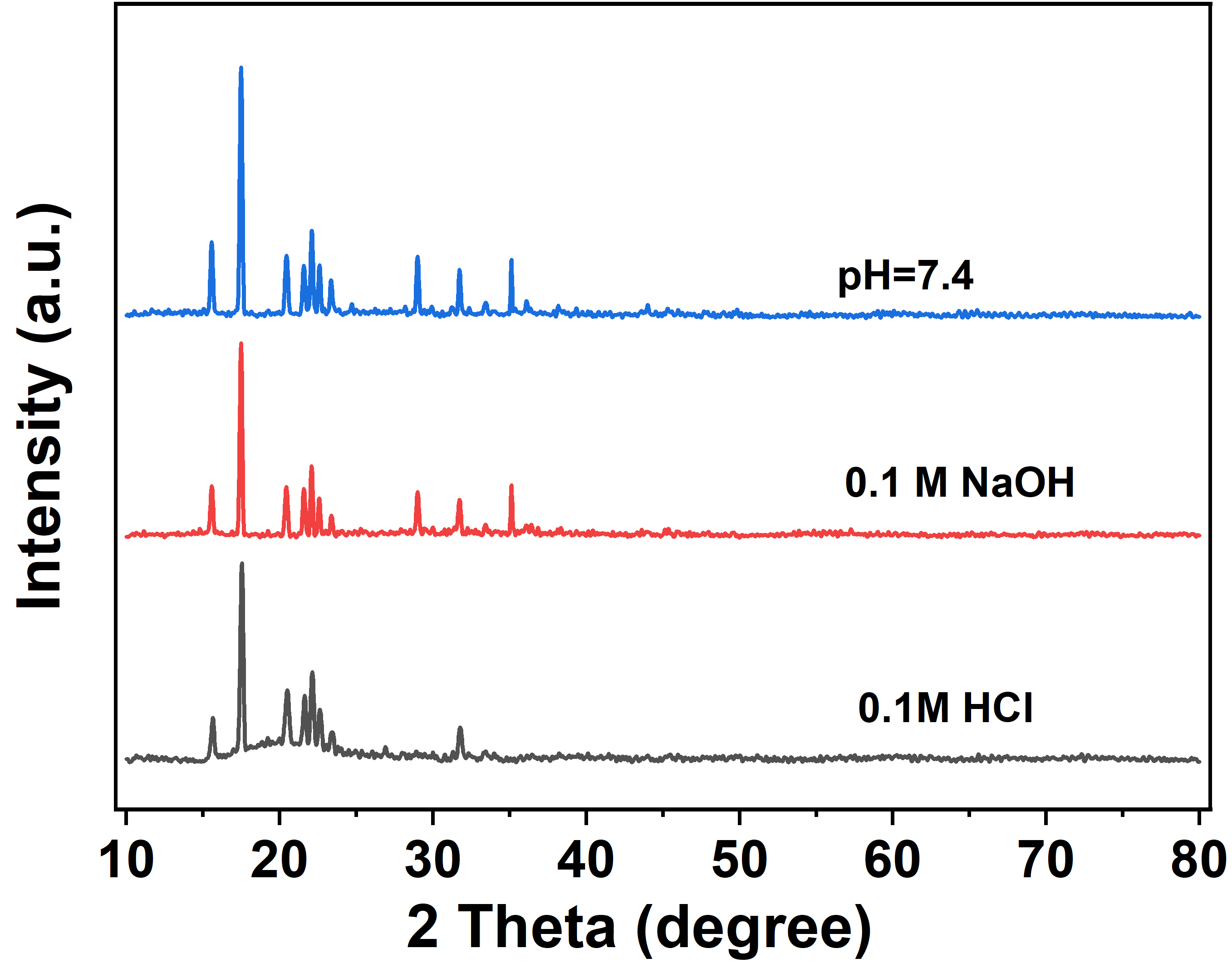


**Fig. S1** XRD patterns the HCF upon treatment in different solvents for 7 days.


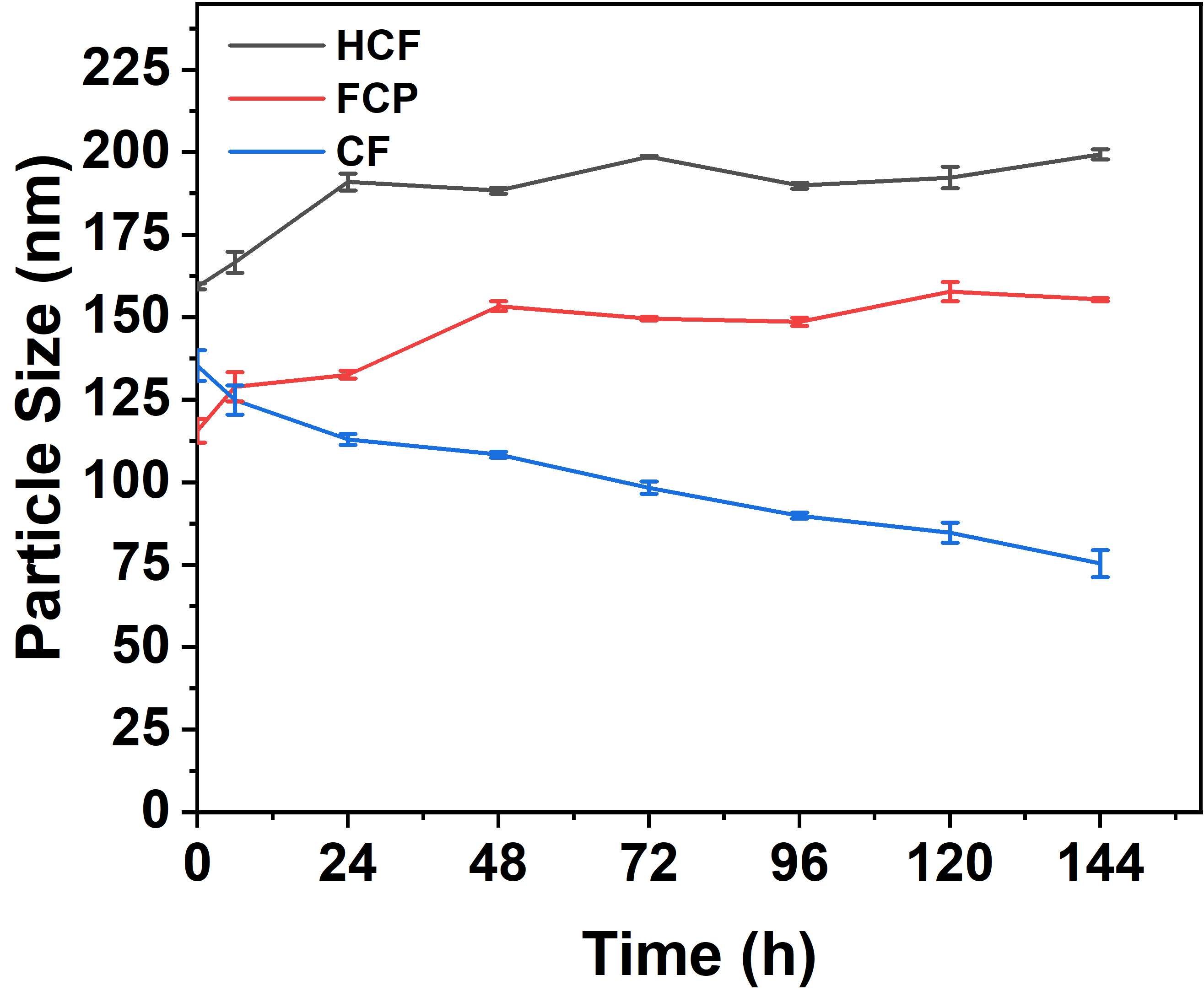


**Fig. S2** Particle size changes of CF, FCP and HCF nanosystem incubated in serum at pH 7.4 for 6 days, detected by DLS.


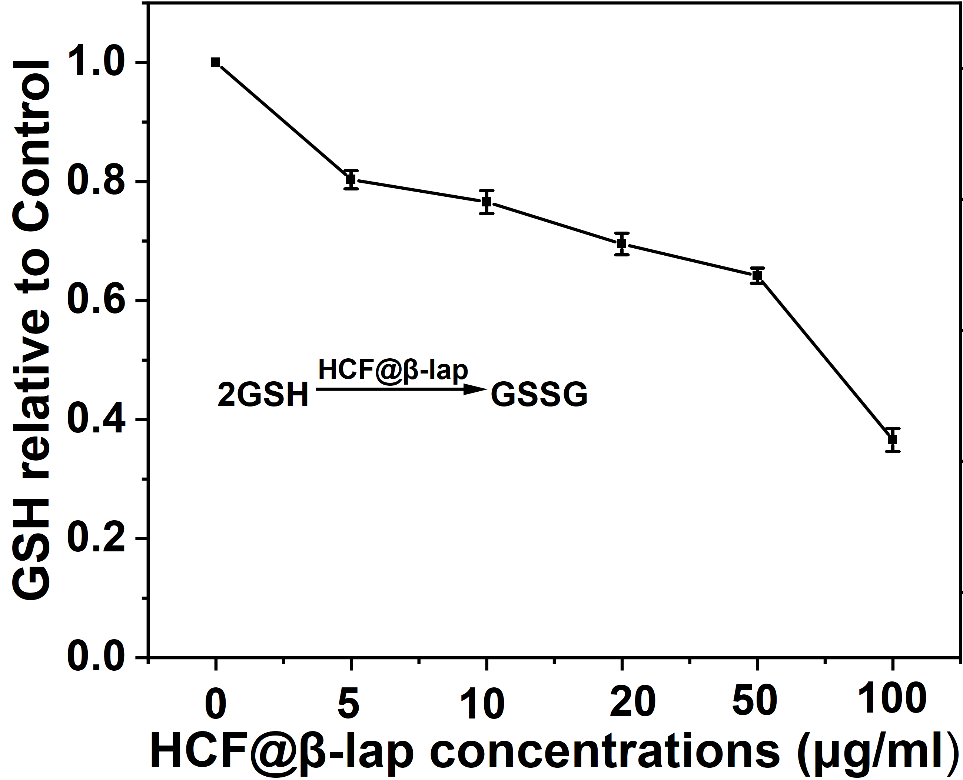


**Fig.** **S3** The amount of GSH in the supernatant after 24 hours of reaction between HCF@β-lap at different concentrations and 10 mM GSH in mixed solution.

**


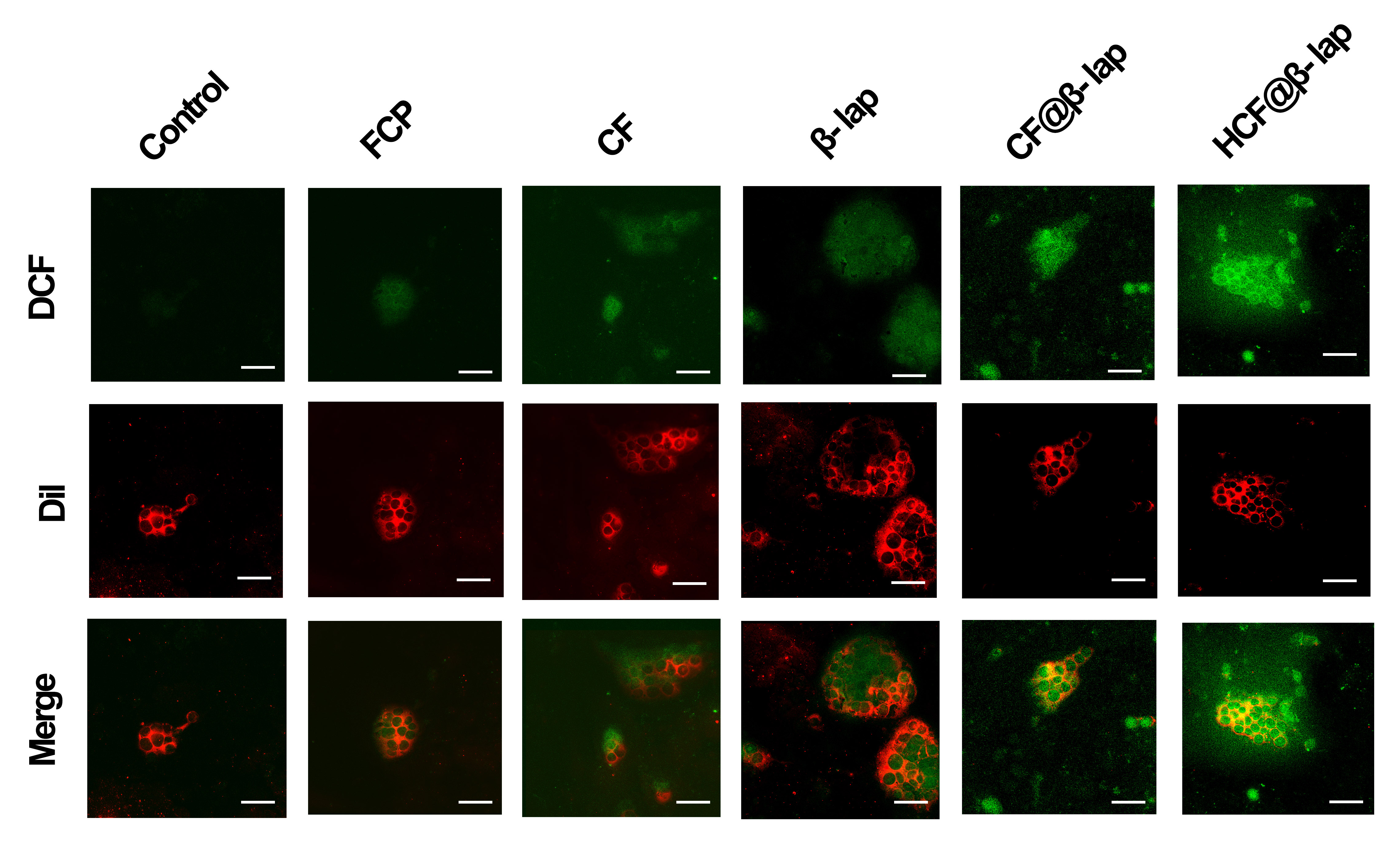
a b**

**Fig. S4** (a) ROS levels in 4T1 cells and cell membrane staining after different treatments detected by CLSM. (b) The corresponding quantitative analysis via Image J software. Scale bar:50 μm.

**
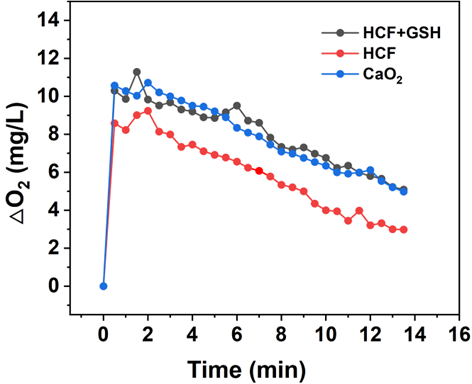
**

**Fig. S5** O_2_ concentration measurement behavior of various nanoparticles in the presence of the weak acidic microenvironment (pH = 6.8, CaO_2_ =100 μg mL^−1^).


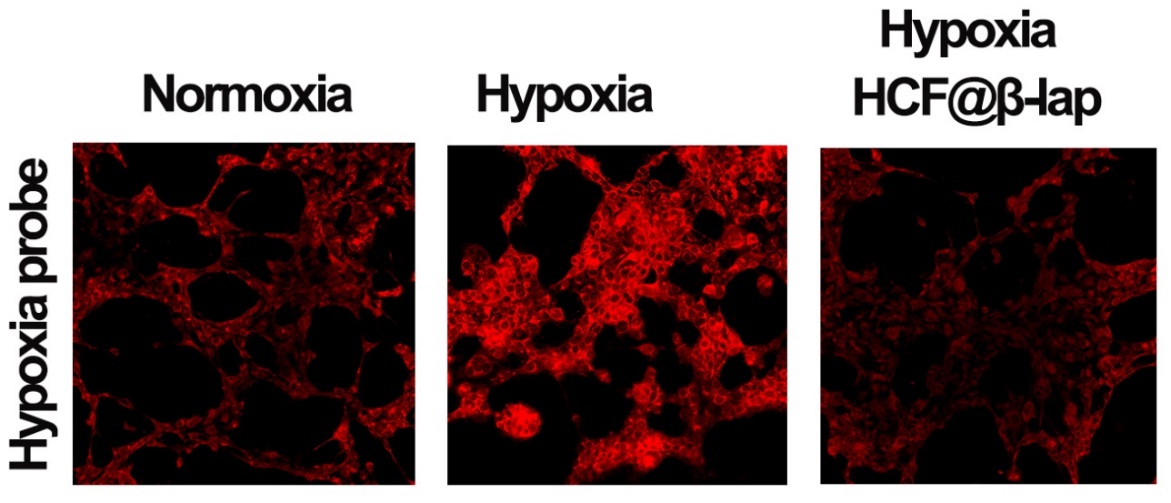


**Fig.** **S6** CLSM images of hypoxia level in 4T1 cells after PBS and HCF@β nanosystem treatment for 4 h.

**
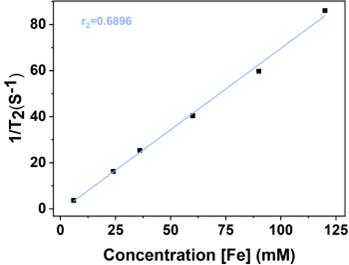

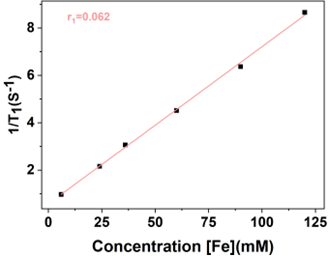
**

**Fig. S7** Relaxation rates *r1* and *r2* of solutions of the HCF@β-lap.


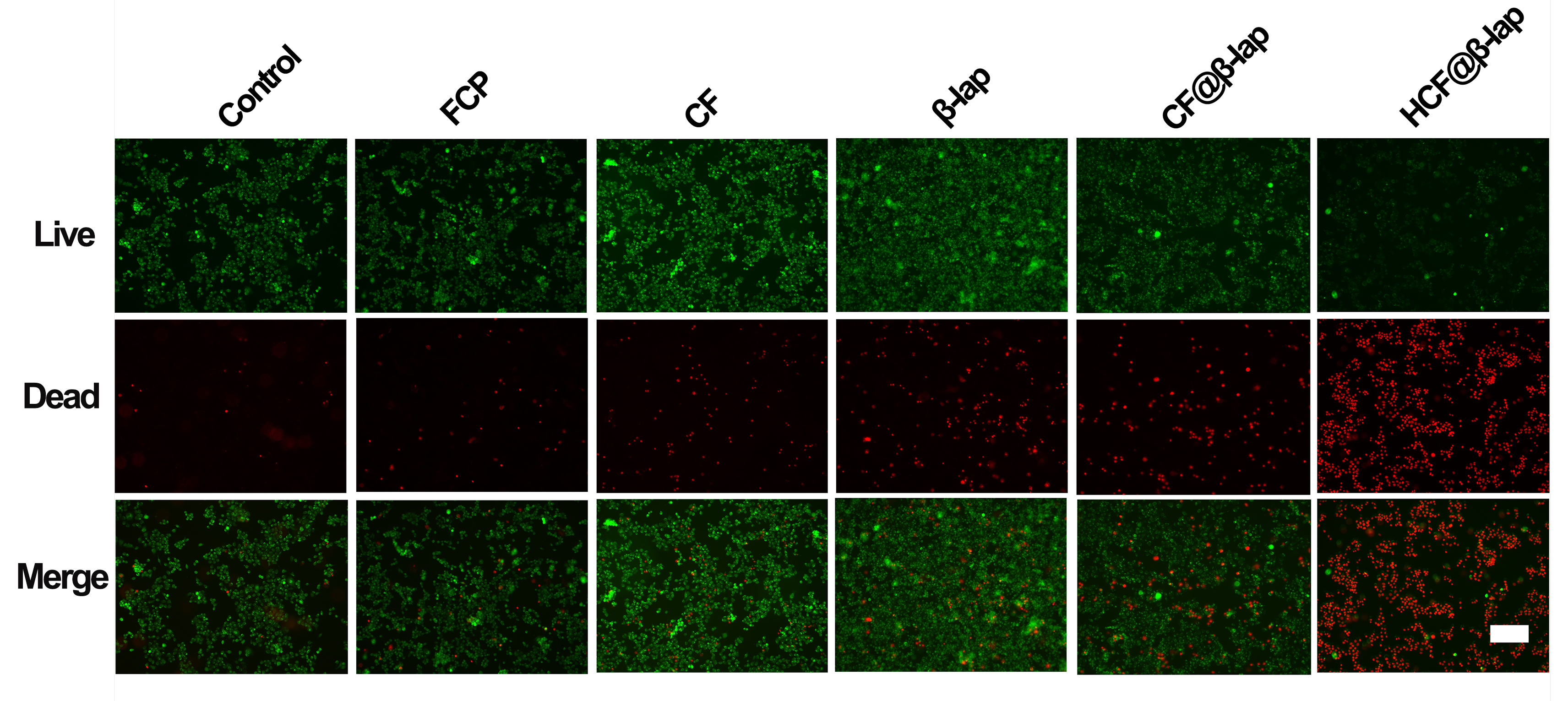


**Fig. S8** 4T1 cell live/dead staining after various treatments. The red signal denoted dead cells, while the green signal denoted live cells. Scale bar:100 μm.


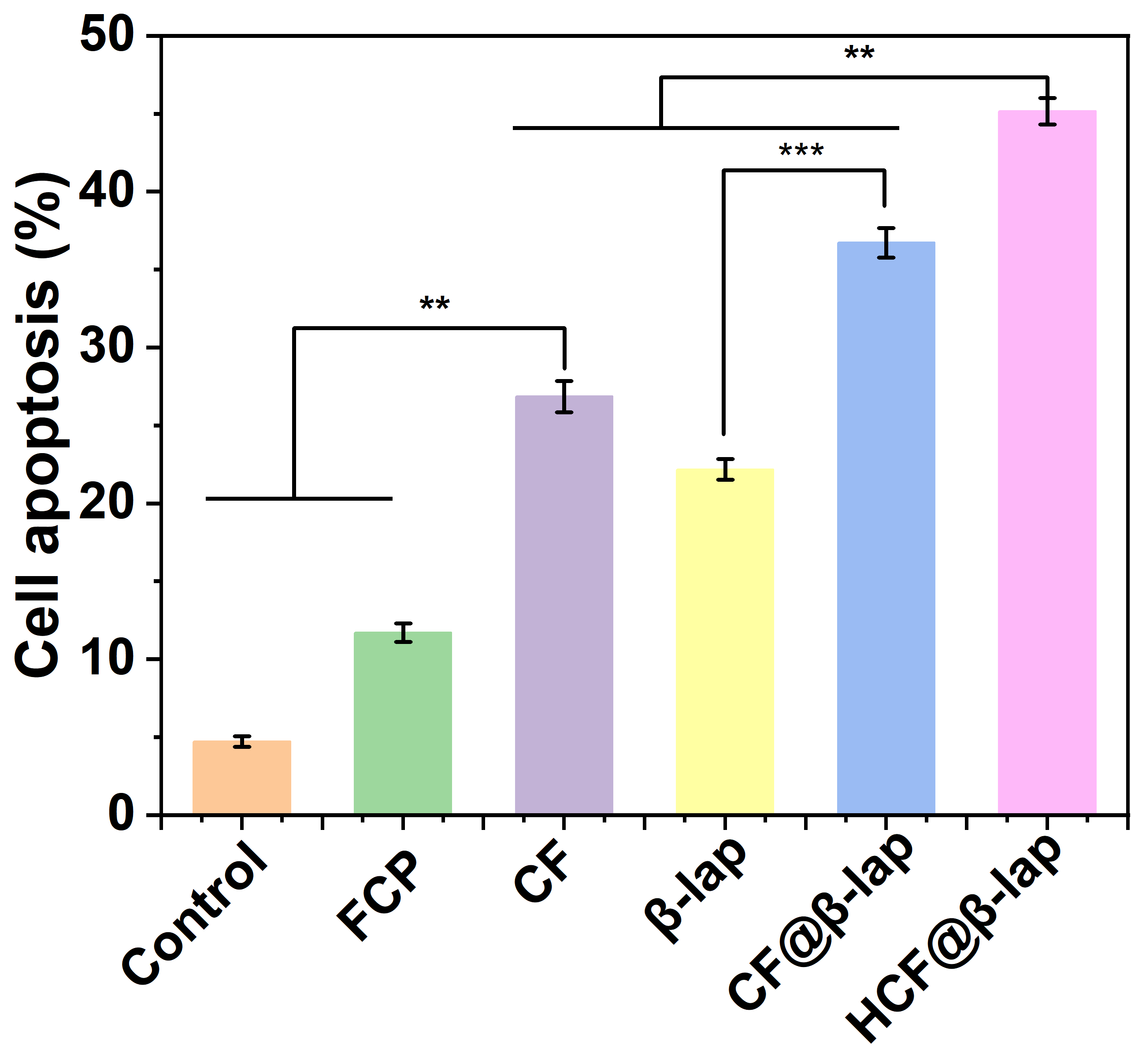


**Fig.** **S9** Quantitative apoptosis statistics of 4T1 cells induced by difference treatments after 24 h of incubation (n=4).


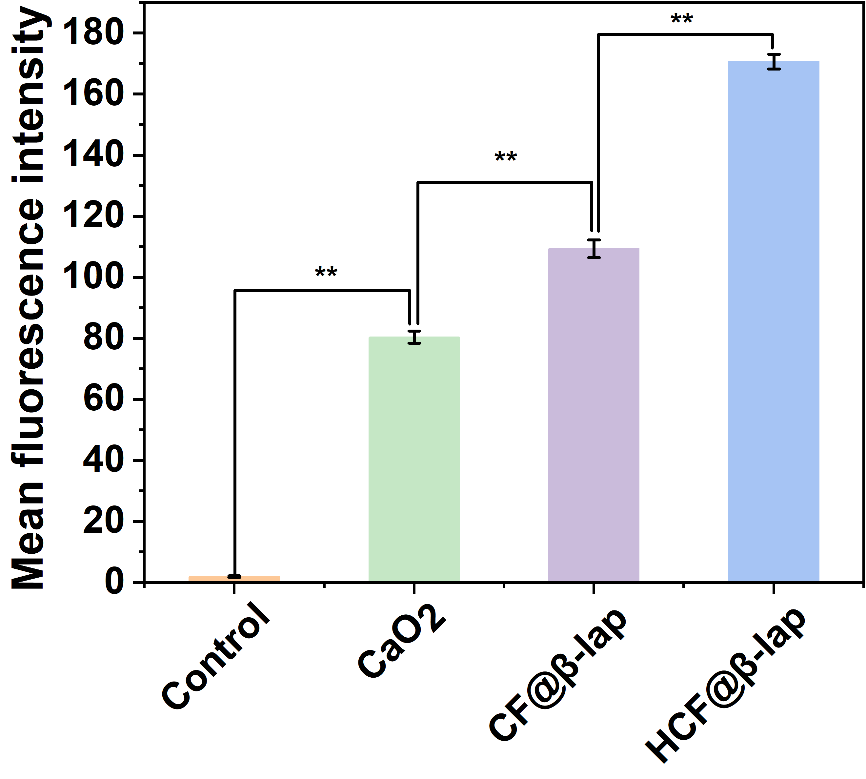


**Fig. S10** The corresponding quantitative analysis of intracellular Ca^2+^ concentration via Fluo-4, AM staining.


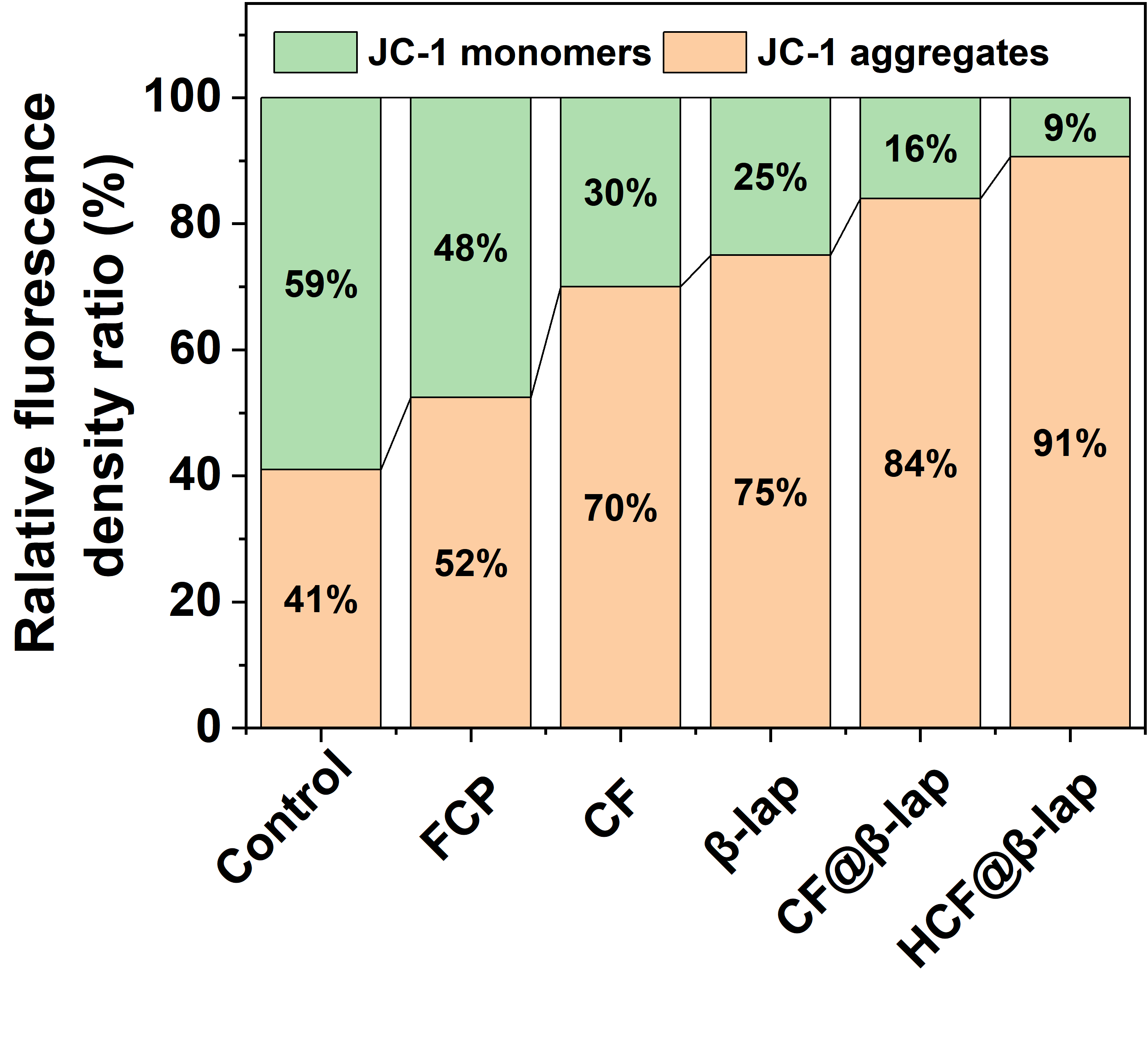


**Fig. S11** The relative fluorescence density analysis of 4T1 cells on mitochondrial damage after administration for 12 h using JC-1 probe.

**
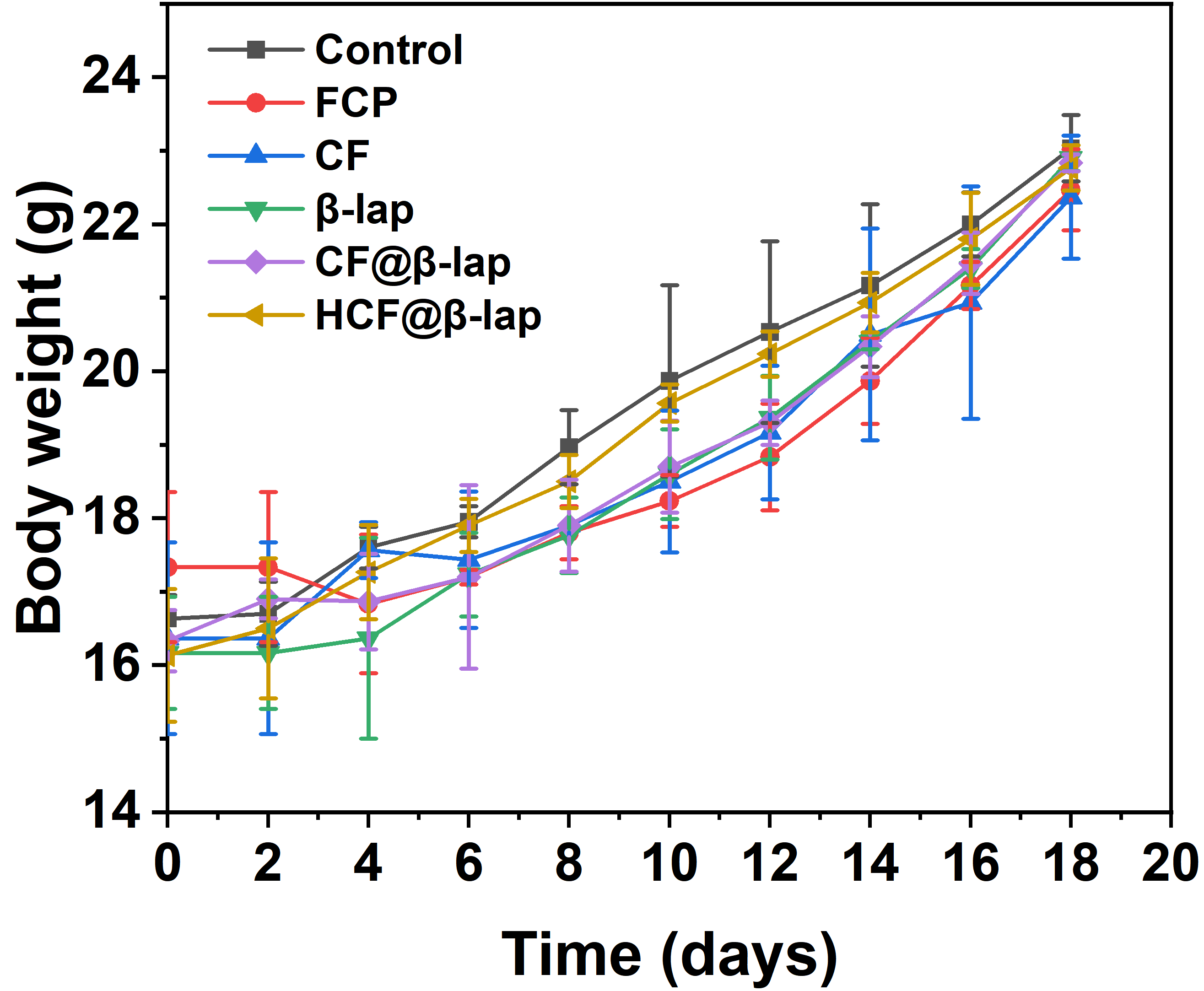
**

**Fig.** **S12** Changes in mice body weight during administration.


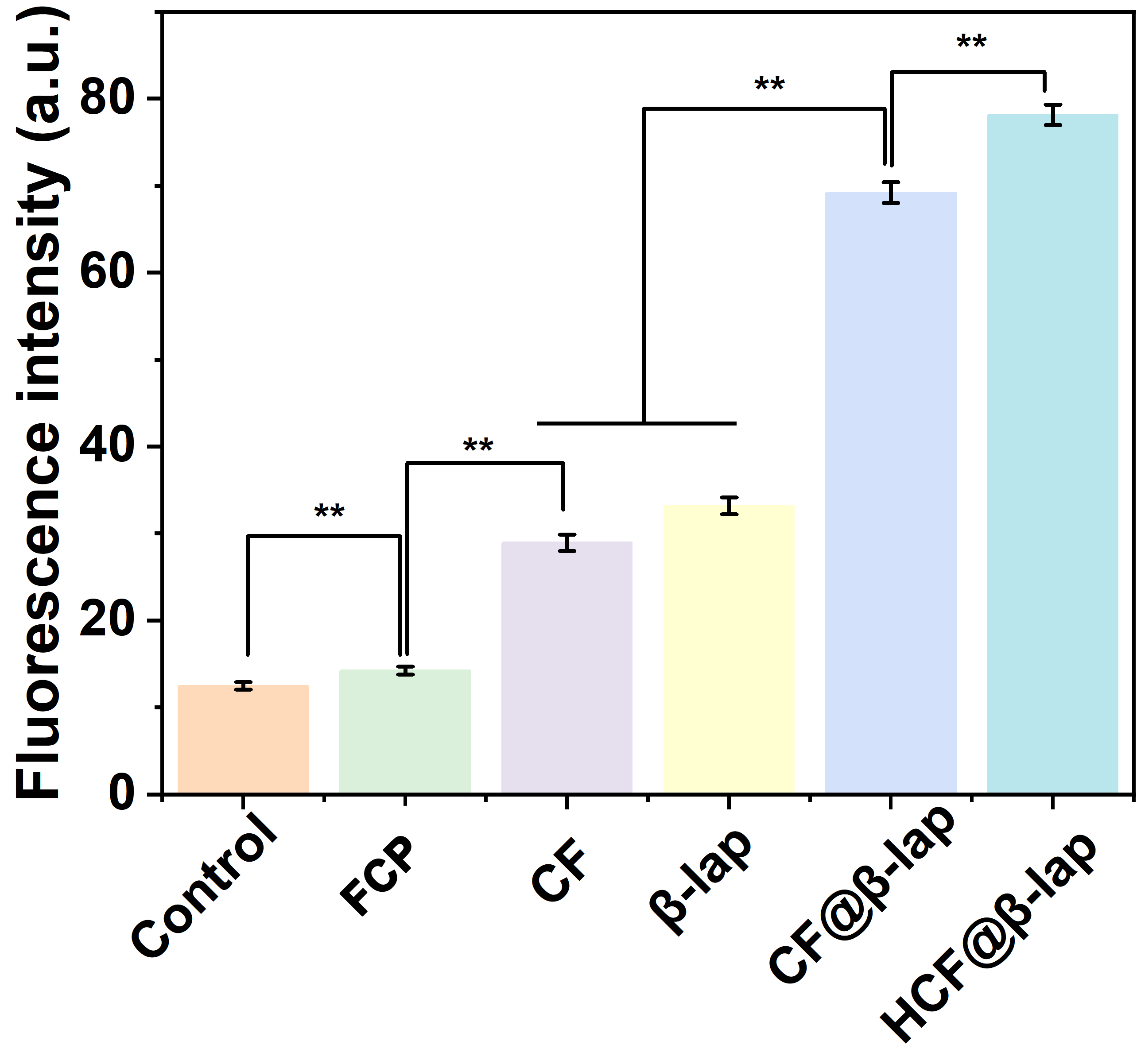

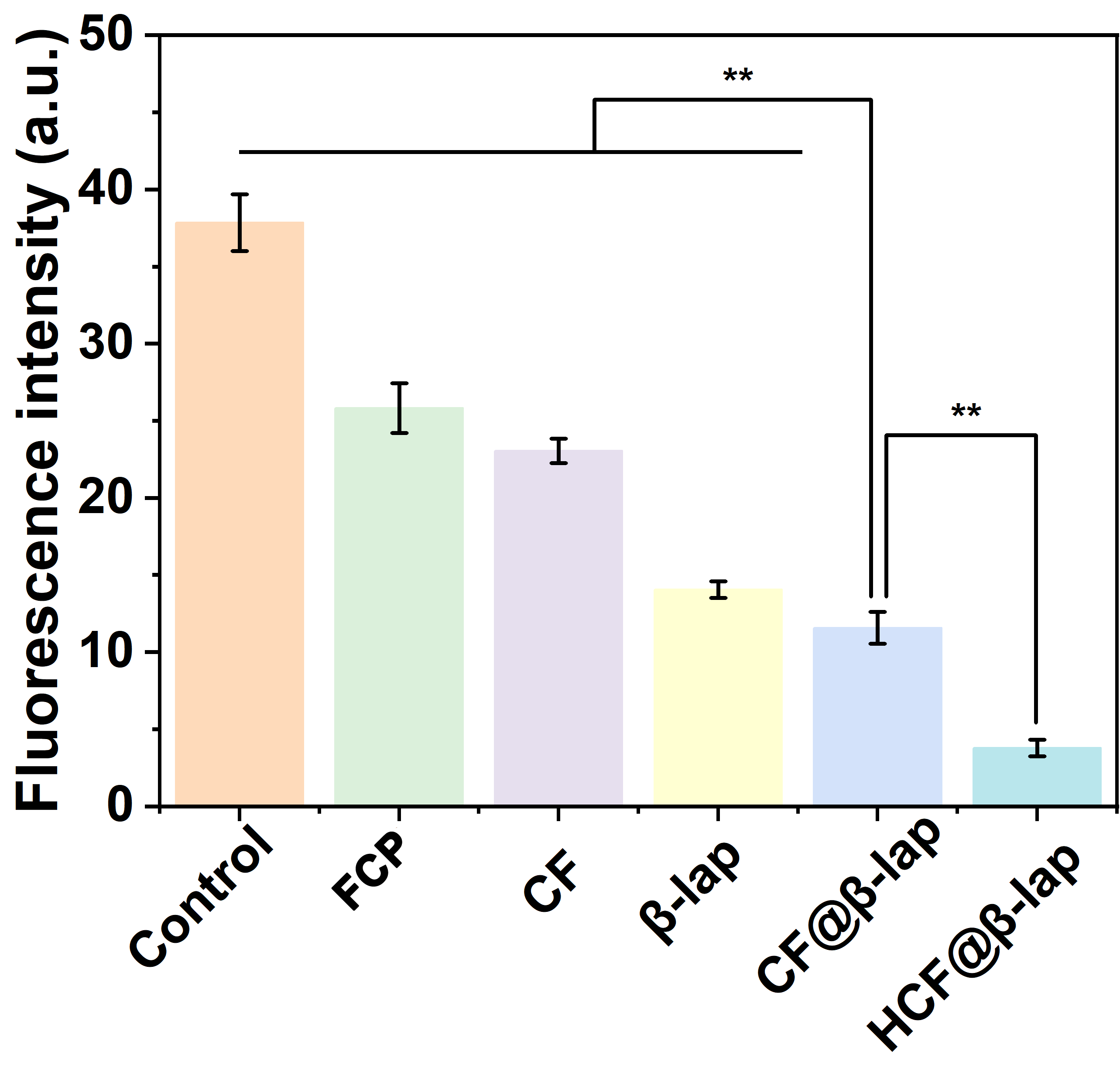


**Fig.** **S13** Tumor-related quantitative analysis of TUNEL and Ki67 immunofluorescence images, Significance analysis was performed using a one-way ANOVA, **p < 0.01 and ***p < 0.001.


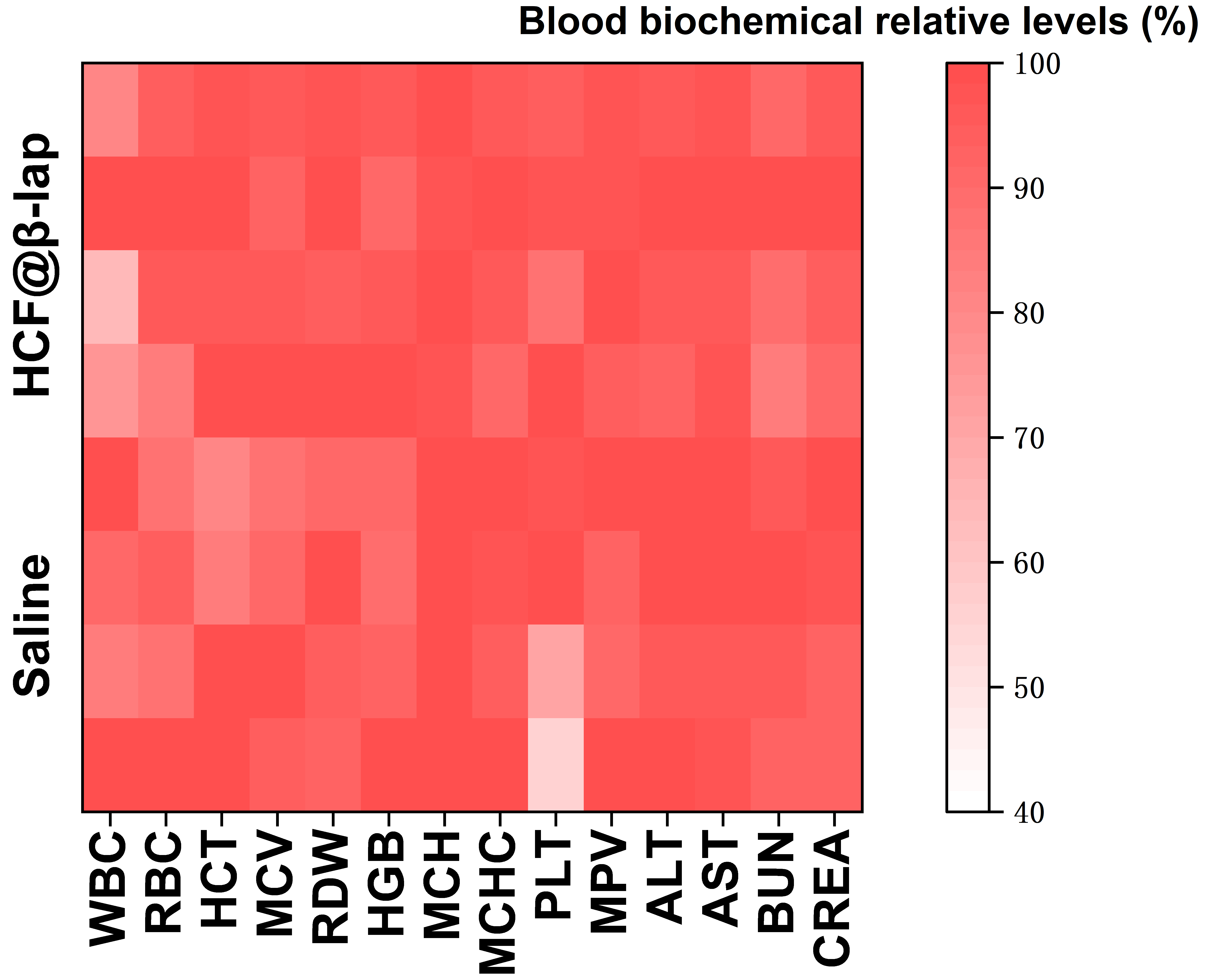


**Fig. S14** The blood biochemical levels and hematological indices of mice after 7 day of administration.


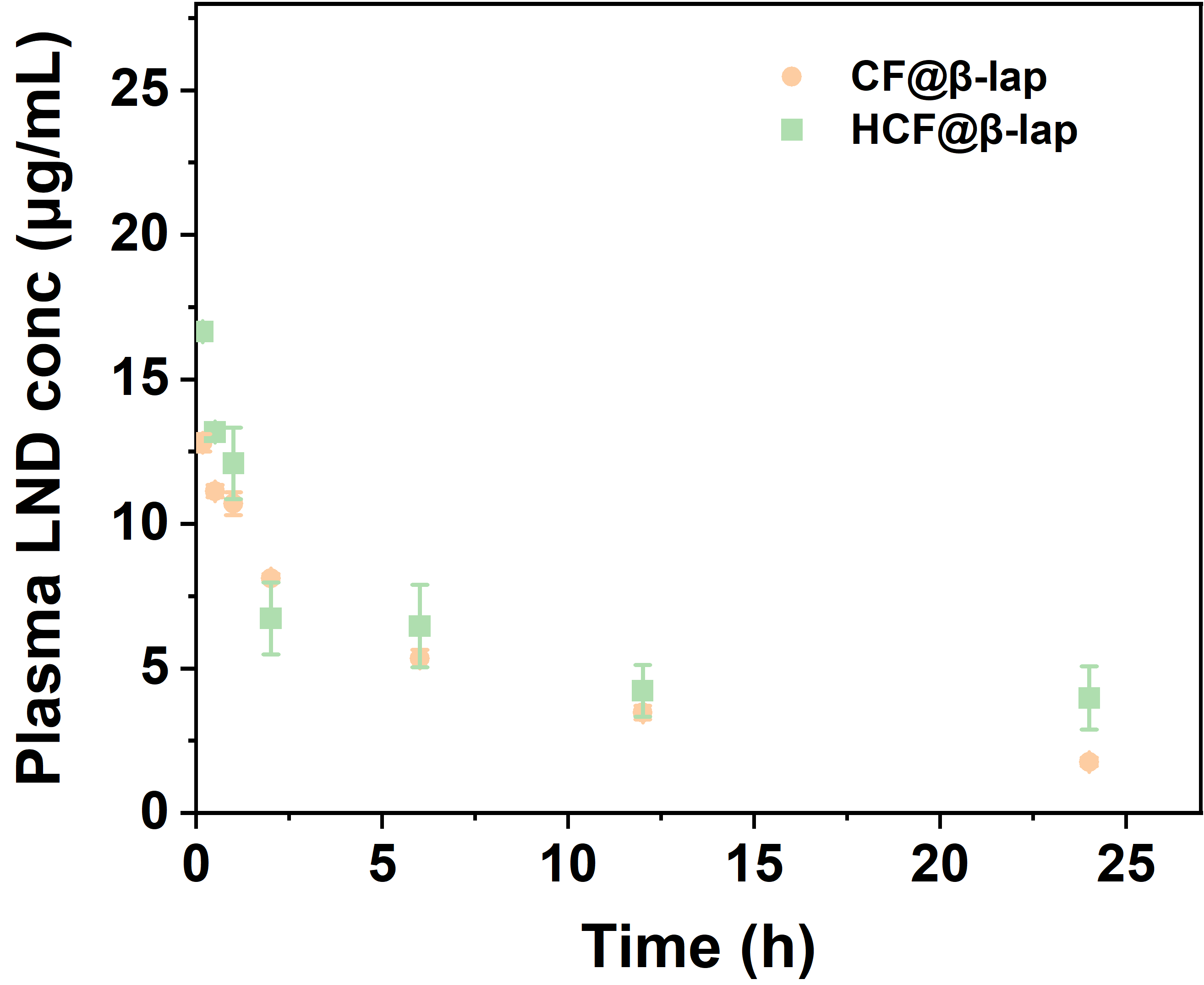


**Fig. S15** The pharmacokinetics of HCF@β-lap and CF@β-lap after intravenous injection into tumor-bearing mice for 24 h.

| Group | Hydrated particle size | PDI |
| --- | --- | --- |
| FCP | 119.5±3.26 | 0.140 |
| CaO_2_ | 87.57±8.96 | 0.277 |
| CF | 136.47±4.78 | 0.208 |
| HCF | 166.52±4.21 | 0.182 |
| FCP+GSH | 2101.14±19.17 | 0.218 |
| HCF+GSH | 1672±32.15 | 0.236 |

**Table S1**. Hydrated particle size and dispersion coefficient of each group.

| Ratio (CaO_2_/FCP) | DLS | Zeta potential (mV) | PDI |
| --- | --- | --- | --- |
| 1:10 | 416.3±3.56 | -46.91 | 0.230 |
| 1:33 | 518.21±8.3 | -41.80 | 0.251 |
| 1:2 | 463±3.32 | -30.23 | 0.254 |
| 1:1 | 133.03±2.96 | -20.13 | 0.197 |

**Table S2**. Hydrated particle size, zeta potential and PDI at different mass ratios between CaO_2_ and FCP.
